# A comprehensive review of ethnomedicinal approaches, phytochemical analysis, and pharmacological potential of *Vitex trifolia* L.

**DOI:** 10.3389/fphar.2024.1322083

**Published:** 2024-03-21

**Authors:** Javad Mottaghipisheh, Marzie Kamali, Amir Hossein Doustimotlagh, Mohammad Hossein Nowroozzadeh, Fatemeh Rasekh, Mohammad Hashem Hashempur, Aida Iraji

**Affiliations:** ^1^ Research Center for Traditional Medicine and History of Medicine, Department of Persian Medicine, School of Medicine, Shiraz University of Medical Sciences, Shiraz, Iran; ^2^ Molecular Medicine Research Center, Hormozgan Health Institute, Hormozgan University of Medical Sciences, Bandar Abbas, Iran; ^3^ Department of Clinical Biochemistry, Faculty of Medicine, Yasuj University of Medical Sciences, Yasuj, Iran; ^4^ Medicinal Plants Research Center, Yasuj University of Medical Sciences, Yasuj, Iran; ^5^ Ophthalmology Research Center, Department of Ophthalmology, School of Medicine, Shiraz University of Medical Sciences, Shiraz, Iran; ^6^ Department of Biology, Payame Noor University (PNU), Tehran, Iran; ^7^ Stem Cells Technology Research Center, Shiraz University of Medical Sciences, Shiraz, Iran

**Keywords:** *Vitex trifolia* L., secondary metabolites, ethnomedicine, phytochemistry, bioactivities

## Abstract

Plants, renowned for their rich reservoir of metabolites, play a pivotal role in addressing health-related issues. The Verbenaceae family stands out, showcasing immense potential in preventing and treating chronic diseases. *Vitex trifolia* L. (*V. trifolia*), a shrub with a rich history in traditional medicine, particularly in Eastern Asia, has garnered attention for its diverse therapeutic applications. This comprehensive review aims to bridge traditional knowledge and contemporary insights by investigating ethnopharmacology, phytochemistry, and pharmacological effects of *V. trifolia*. The keyword “*V. trifolia*” and its synonyms were searched within the main scientific databases including PubMed, Web of Science, ScienceDirect, Google Scholar, and Baidu Scholar (from 1974 to 2022, last search: 21.10.2023). Phytochemical analyses reveal a spectrum of secondary metabolites in *V. trifolia*, including terpenoids, flavonoids, lignans, phytosterols, anthraquinones, and fatty acids. Notably, terpenoids and flavonoids emerge as the main bioactive metabolites. Pharmacological studies validate its therapeutic potential, demonstrating significant antioxidant, anti-inflammatory, hepatoprotective, anticancer, anti-amnesic, antimicrobial, antiviral, anti-malaria, antispasmodic activities, and reported insecticidal effects. Despite existing literature exploring pharmacological attributes and secondary metabolites of related species, a conspicuous gap exists, specifically focusing on the pharmacological activities and novel methods of purification of pure metabolites from *V. trifolia*. This review aimed to fill this gap by delving into traditional medicinal applications, exploring secondary metabolites comprehensively, and providing an in-depth analysis of pharmacological effects of pure metabolites. Combining traditional uses with contemporary pharmacological insights, this article sought to serve as a crucial reference for future research and practical application of *V. trifolia*. This approach contributes substantially to understanding the plant, fostering scientific inquiry, and facilitating its broader application in healthcare.

## 1 Introduction

Plants play a crucial role in health-related problems due to their rich reservoir of bioactive metabolites. These natural metabolites found in various plant species, such as those belonging to the Verbenaceae family, have shown immense potential in preventing and treating chronic diseases, offering a promising opportunity for therapeutic interventions (Hashempur et al., 2018; Alyasin et al., 2020; Conti et al., 2021). Verbenaceae is one of the largest families of the plant kingdom, consisting of trees, shrubs, lianas, and herbs. Verbenaceae comprise of 34 genera and around 1,200 species. *Vitex* is known as one of the largest genera in the family, possessing 270 species mainly distributed in tropical areas, with a few in subtropical regions (Rani and Sharma, 2013; Yao et al., 2016).

*Vitex trifolia* L. (*V. trifolia*) is a shrub or shrubby tree that may grow up to 6 m in height. It is found in some regions of Asia, China, India, Indonesia, Sri Lanka, Singapore, and Australia. This plant has a rich history in traditional medicine for its effectiveness in treating asthma and respiratory disorders. It has been reported that most plant parts such as the fruit, leaf, root, flower, and stem demonstrated medicinal values; however, its fruit is the most studied and used part (Zaki et al., 2022). Different types of secondary metabolites including terpenoids (mainly labdane-type diterpenes), flavonoids, lignans, phytosterols, anthraquinones, and fatty acids have been reported in *V. trifolia*; whereas terpenoids and flavonoids have been identified as the main bioactive compounds (Yan et al., 2023). Pharmacological studies have further validated its therapeutic potential by demonstrating significant antioxidant, anti-inflammatory, hepatoprotective, anticancer, anti-amnesic, antimicrobial, antiviral, anti-malaria, and antispasmodic activities. Additionally, some studies have reported its insecticidal effects (Tandon et al., 2008). These findings highlight the diverse beneficial properties associated with *V. trifolia* and support its use in traditional medicine for various health conditions.

In recent years, the medicinal potential of *V. trifolia* has garnered significant attention, particularly within traditional medicine. A series of review papers have explored various aspects of this plant species with emphasis on its pharmacological properties (Chan et al., 2016; Kamal et al., 2022; Yan et al., 2023). The most recent review explores the pharmacological attributes and secondary metabolites of *V. trifolia* L. and *V. rotundifolia* L. f., providing insights into their properties. However, despite the existing body of literature, there remains a conspicuous gap in the current discourse, particularly the phytochemical aspects of *V. trifolia*, as an invaluable natural agent, where complementary information regarding the plant parts and extracts utilized, also different separation methodologies could indeed assist futuristic investigations of this species.

The current study is an attempt to fill this gap by investigating the traditional medicine applications of *V. trifolia*, along with an in-depth analysis of its secondary metabolites through an updated literature search strategy, where existing information on chromatographic steps and the plant parts/extracts employed which led to the isolation and identification of its precious secondary metabolites are described in detail.

Moreover, it explores the pharmacological effects of the individual pure metabolites isolated from *V. trifolia* a crucial aspect that has thus far been overlooked in the existing literature. Hence, this article aimed to serve as a vital reference for future research endeavours and the practical utilization of *V. trifolia* Through a comprehensive exploration of both the traditional uses and contemporary pharmacological insights, this review is an attempt to contribute substantially to understanding *V. trifolia* and pave the way for further scientific inquiry and application of this intriguing plant species.

## 2 Methodology

In this study, a comprehensive literature search was done focusing on *V. trifolia* across various online databases and relevant books. The search employed the term ‘*Vitex trifolia*’ and its synonyms ‘*Vitex agnus-castus* var. *trifolia* (L.) Kurz, *Vitex indica* Mill., *Vitex integerrima* Mill., *Vitex trifolia* var. *trifoliolata* Schauer, and *Vitex variifolia* Salisb.’, (confirmed by http://www.plantsoftheworldonline.org), while targeted prominent databases including PubMed, Web of Science, ScienceDirect, and Google Scholar. Baidu Scholar were also included in the search with a specified time frame from 1974 to 2022 (the last search was conducted on 21.10.2023). The search yielded 889, 283, 1,263, 1,023, and 147 articles in each database, respectively. After this refinement process, a total of 164 articles emerged as pertinent to the scope of this review. This judicious selection ensures that the information is comprehensive and focused, contributing to the literature review robustness.

## 3 Traditional uses of *V. trifolia*

Various ethnopharmacological studies have demonstrated promising medicinal applications for different plants (Mosavat et al., 2015; Ayeni et al., 2022; Das et al., 2022). *V. trifolia*, commonly known as the chaste tree or five-leaved chaste tree, has gained recognition as a botanical drug, particularly in Eastern Asia. Throughout the years, ethnomedicinal investigations have documented the diverse therapeutic applications of this plant (Blois, 1958; Ban et al., 2020).

The traditional use of the fruit can be traced back to ancient times, with its earliest recorded mention appearing in Shen Nong’s Classic of Materia Medica of China. This historical text was commended for its medicinal properties and addressed as a remedy for various afflictions. Among its benefits, it was believed that it alleviated conditions like cold and heat between the tendons and bones, addressed dampness impediment, enhanced vision by brightening the eyes, strengthened the teeth, unblocked the “nine orifices” (body openings), and even eliminated taeniasis, a condition caused by tapeworm infection. This valuable fruit has a significant place in traditional medicine, connecting its potent healing qualities to improve human health (Wu et al., 2009a). Furthermore, it has been credited with promoting hair growth. Tonga, known for its rich traditional practices, utilized the plant for its remarkable healing properties. Specifically, they harnessed its powers to treat oral infections and inflammations (Limousin and Bessières, 2006).

In Unani medicine, the plant is known as Sambhalu and has been employed to reduce libido (Suchitra and Cheriyan, 2018). In Papua New Guinea, the indigenous population utilizes the stem of *V. trifolia* L. to treat dysentery. The leaves of *V. trifolia*, called Jalanirgundi in traditional Ayurvedic medicine, are commonly prepared as a decoction or used topically as a poultice. They have been employed to alleviate joint pain, inflammation, and rheumatism (Kirtikar and Kirtikar, 1980; Thenmozhi et al., 2015). In New Caledonia, Rotuma, and the Solomon Islands, heated leaves are commonly used to alleviate severe headaches by rubbing them on the forehead or consuming them as an infusion (de Kok, 2007). The fruits of *V. trifolia*, commonly called Manjingzi in the Chinese Pharmacopoeia, have a long-standing history in traditional Chinese medicine. They are known for their wind-heat-dispersing properties, making them valuable in treating various ailments such as headaches, migraines, and ophthalmodynia. The traditional use of Manjingzi highlights its effectiveness in addressing conditions associated with wind heat, providing relief, and promoting overall wellbeing. Besides, the flowers of *V. trifolia* have demonstrated usefulness in treating fever (Talreja and Tiwari, 2020).

## 4 Phytochemistry

**FIGURE 1:**
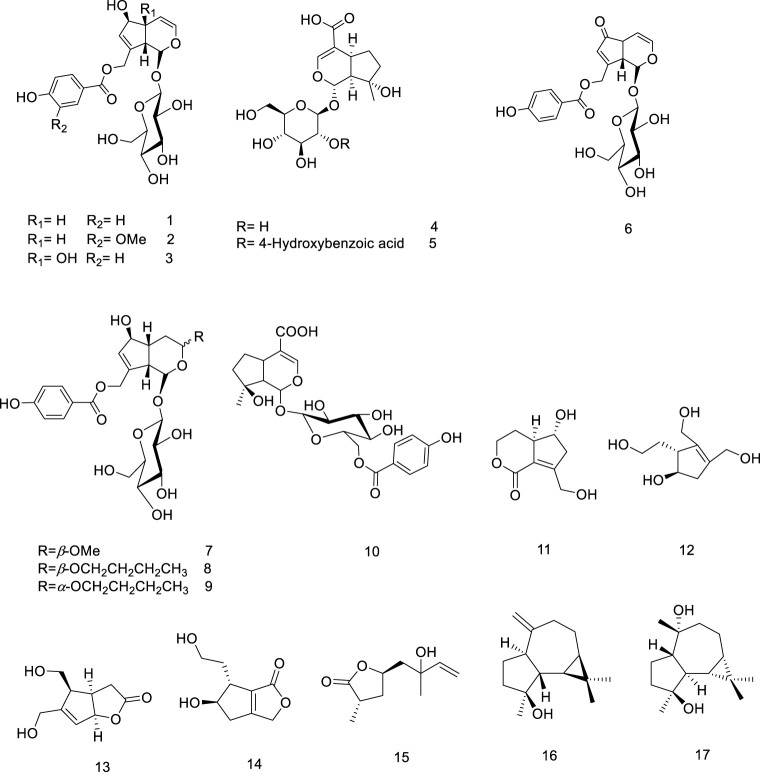
Chemical structure of monoterpenoids and sesquiterpenoids isolated from *V. trifolia.*

**FIGURE 2:**
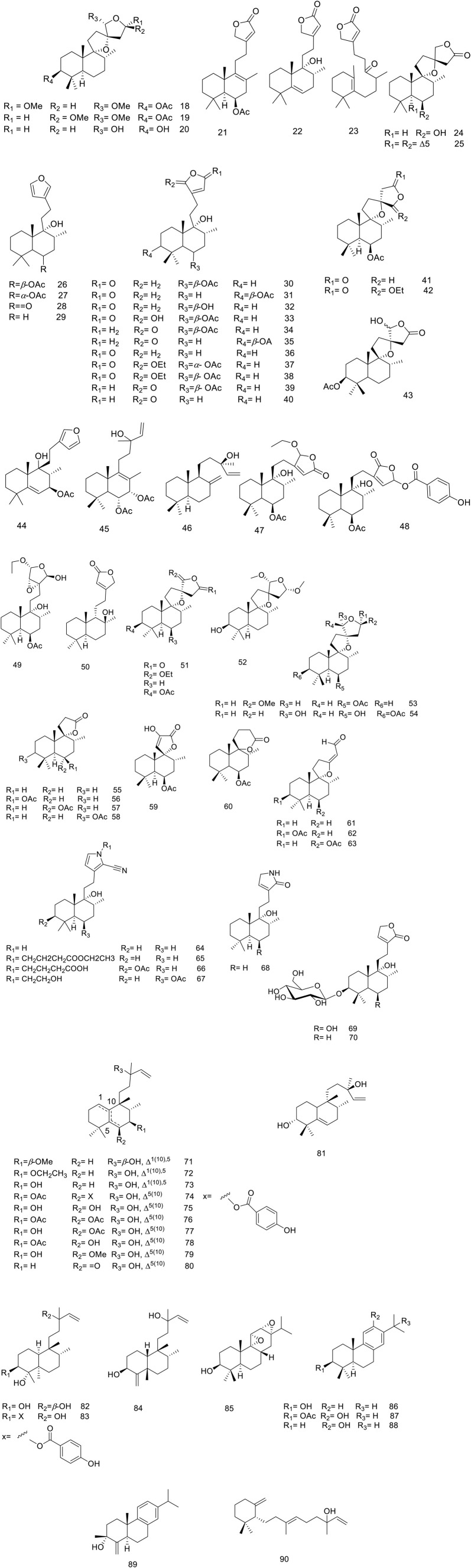
Chemical structure of diterpenoids isolated from *V. trifolia*.

**FIGURE 3:**
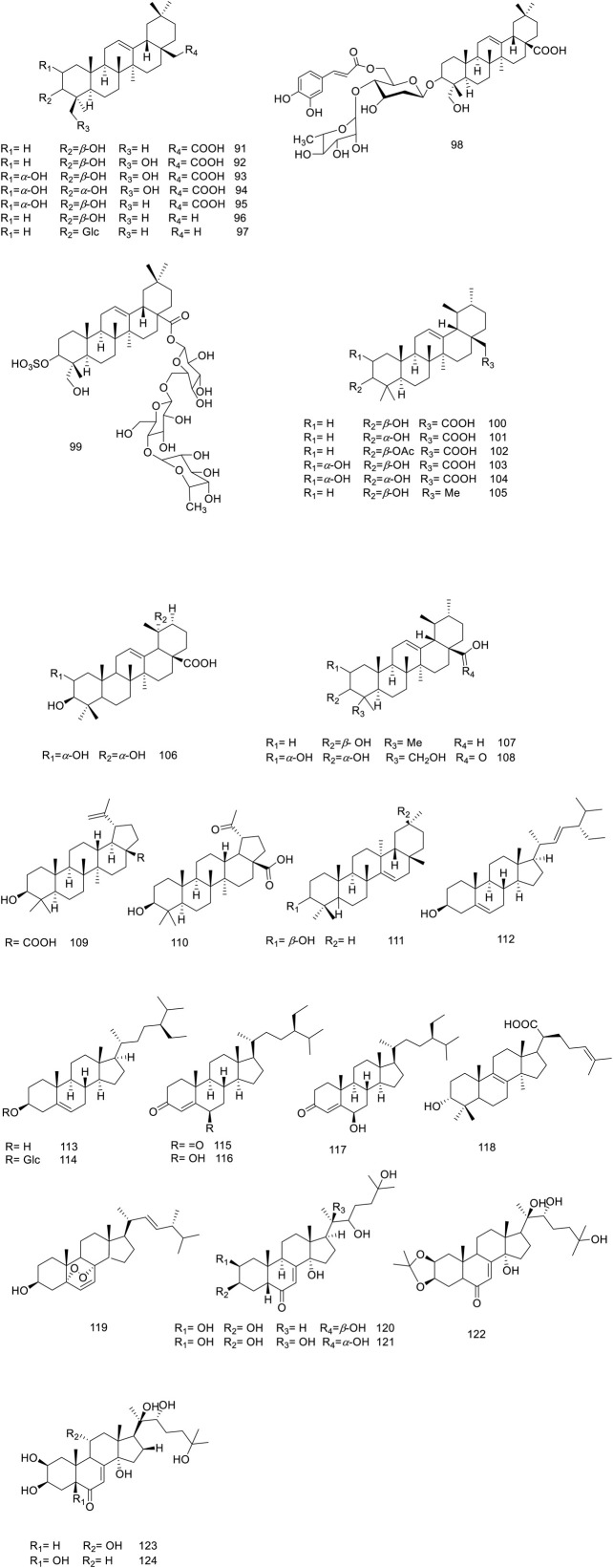
Chemical structure of triterpenoids and phytosterols isolated from *V. trifolia*.

**FIGURE 4:**
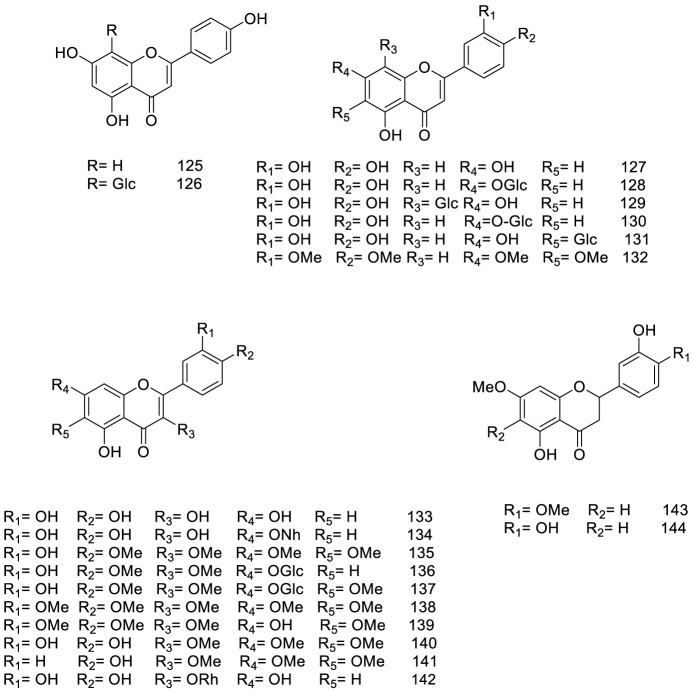
Chemical structure of flavonoids isolated from *V. trifolia*.

**FIGURE 5:**
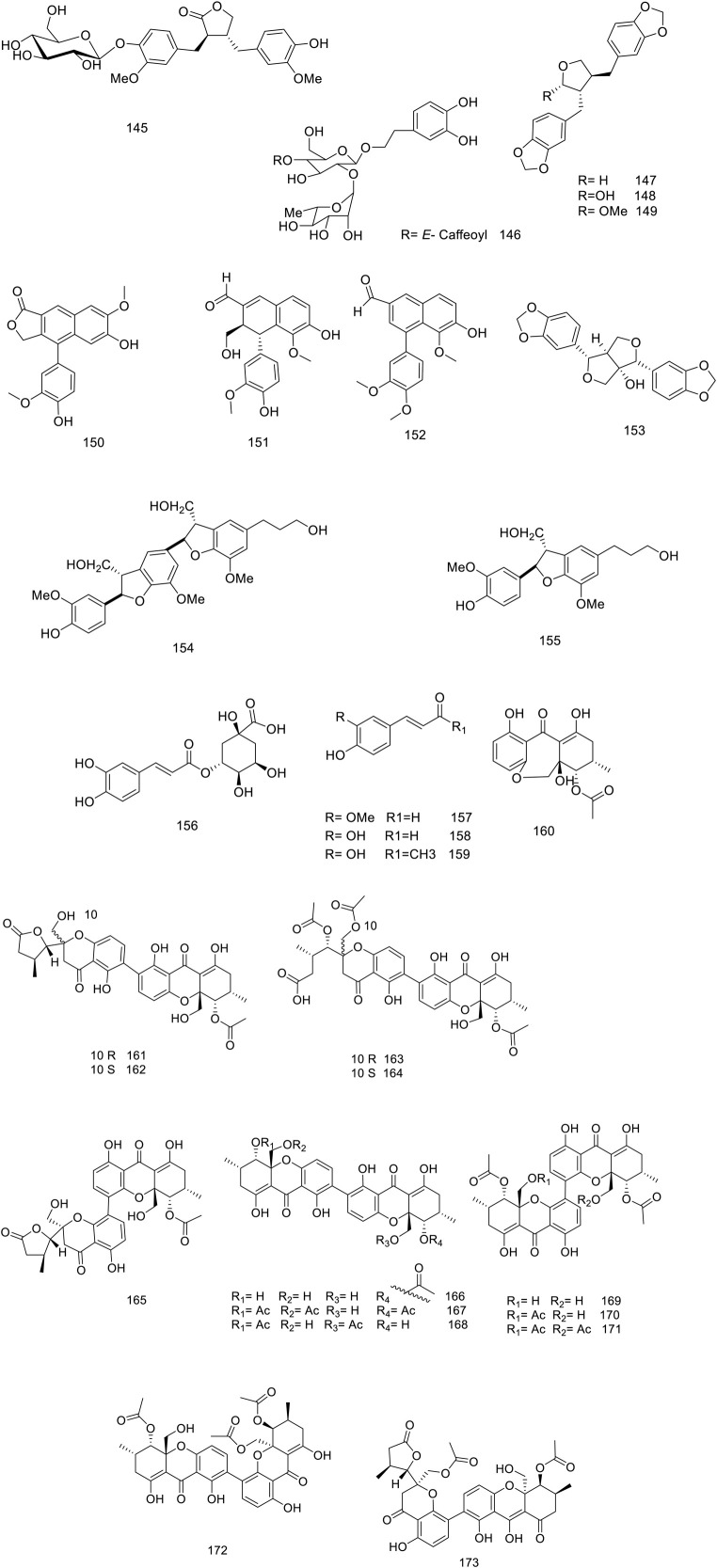
Chemical structure of lignans, phenylpropanoids and xanthones isolated from *V. trifolia*.

**FIGURE 6:**
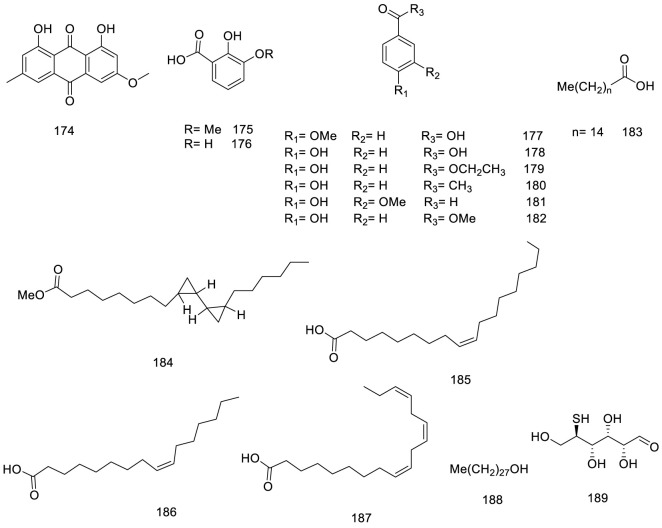
Chemical structure of Miscellaneous metabolites isolated from *V. trifolia*.

**FIGURE 7:**
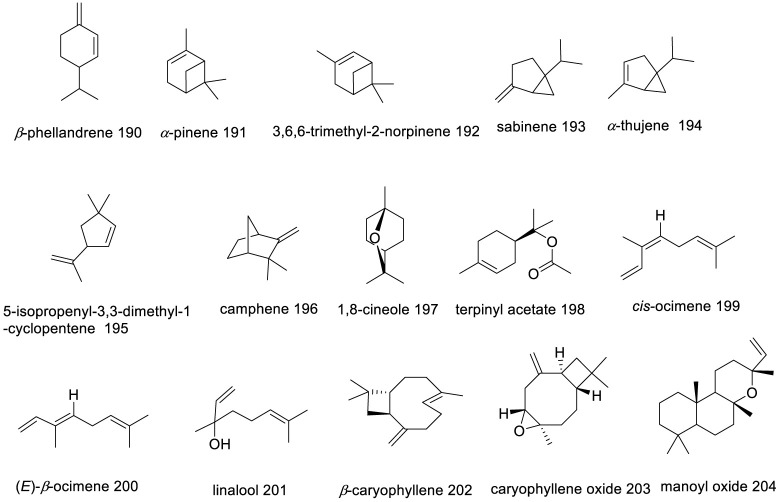
Chemical structures of the major secondary metabolites from *V. trifolia* essential oil.

So far, over 180 metabolites have been identified from different parts of *V. trifolia*. Investigation of the chemical profile has led to the isolation of terpenoids (monoterpenes, sesquiterpenes, diterpenes, triterpenes, and phytosterols), ecdysteroids, flavonoids, lignans, phenylpropanoids, anthraquinone, fatty acids, along with xanthones isolated from the endophytic fungi of the fruit (Peng et al., 2021). Among them, the diterpenes special labdane-type are the most significant metabolites in this species. In the following sections, the isolated/identified phytochemicals have been classified based on their main classifications (Figures 1–7); however, more details including plant parts used and chromatographic techniques applied are listed in Supplementary Table S1.

## 5 Biosynthesis of terpenoids, flavonoids and iridoids

**FIGURE 8:**
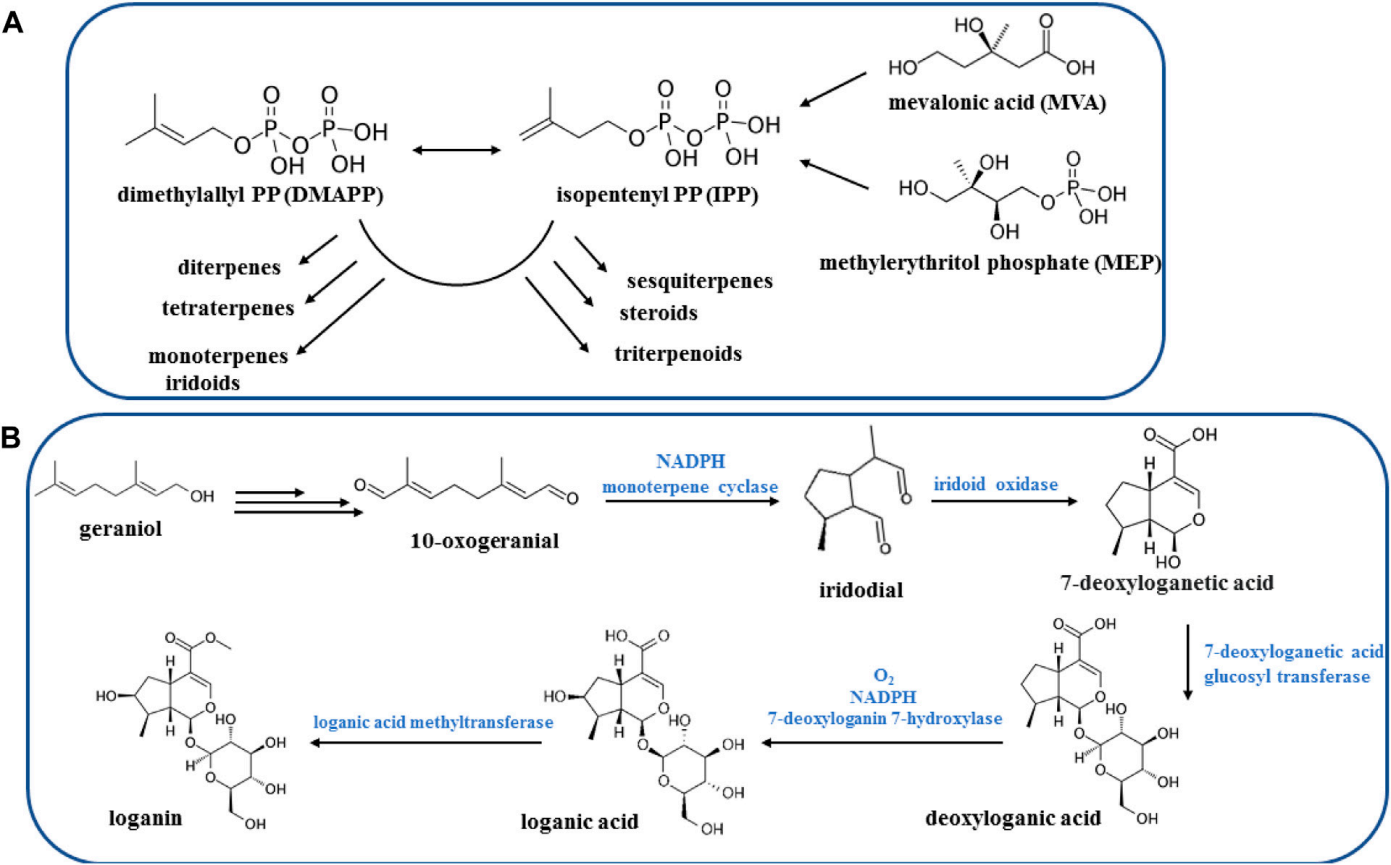
Biosynthesis pathways of terpenoids **(A)** and iridoids **(B)**.

Since the main *Vitex trifolia* compounds identified as terpenoids, flavonoids, and iridoids, thus this section allocates to briefly overview the natural biosynthesis pathways of these compounds. Terpenoids are derived from the mevalonate (MVA) pathway, exhibiting activity within the cytosol, or alternatively from the plasticidal 2-*C*-methyl-D-erythriol 4-phosphate (MEP) pathway. The MEP pathway predominantly serves as the source of hemi-, mono-, di-, and triterpenoids, whereas the MVA pathway is primarily responsible for the synthesis of sesqui- and triterpenoids (Cheng et al., 2007; Maffei et al., 2011) (Figure 8A).

Iridoids represent a vast category of monoterpenoids, distinguished by their skeletal structure consisting of a cyclopentane ring fused with a six-membered ring containing an oxygen atom, commonly known as the iridane skeleton. Typically, these compounds have been identified in plants in conjunction with sugar molecules, rendering their glycosides and allowing for their classification (Villasenor, 2007). The iridoid system is derived from geraniol through a unique folding process, distinct from the folding observed in monoterpenoids. There are over thousand different known natural iridoids, with structural variations primarily arising from hydroxylations, esterifications, and changes in stereochemistry (Figure 8B) (Dewick, 2002).

**FIGURE 9:**
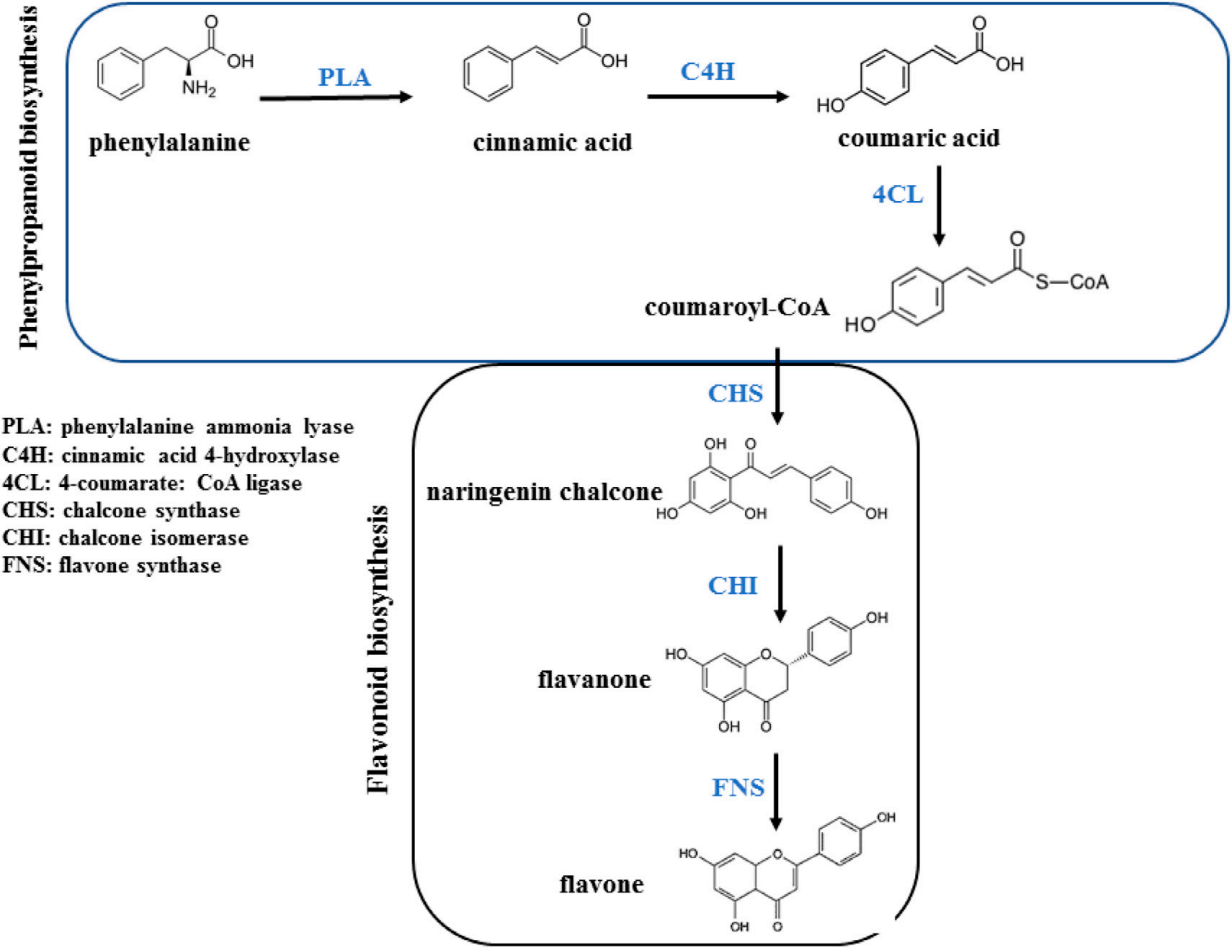
Biosynthesis of flavonoid.

Flavonoids originate from the phenylpropanoid metabolic pathway and possess a fundamental composition consisting of a C15 benzene ring structure of C6-C3-C6. In recent years, substantial research has been conducted to uncover the intricate mechanisms underlying the biosynthesis of flavonoids in plants (Liu et al., 2021). The entry of *p*-coumaroyl-CoA into the flavonoid biosynthesis pathway signifies the start of the synthesis of specific flavonoids, which begins with the formation of chalcone (Nabavi et al., 2020). Chalcone serves as the initial crucial product in the metabolic pathway of flavonoids, offering a fundamental framework for subsequent synthesis of flavonoids (Zhou et al., 2009). Flavanones have the potential to generate numerous iterations based on this fundamental framework, such as flavones, flavonols, anthocyanidins, and catechins (Figure 9) (Mottaghipisheh et al., 2021).

### 5.1 Terpenoids

#### 5.1.1 Monoterpenoids and sesquiterpenoids

Most of the *V. trifolia* monoterpenoids are iridoids and their corresponding glycosides (metabolites **1**–**10**). Besides the iridoids, an acyclic monoterpenoid vitexoid (**15**) has been isolated from the fruits and is a characteristic metabolite of *V. trifolia* (Djimabi et al., 2021). Only two aromadendrane-type sesquiterpenoids (**16** and **17**) were found in the fruits (Gu et al., 2007).

#### 5.1.2 Diterpenoids

The *V. trifolia* fruits and leaves can be considered as a rich source of cyclic diterpenes, predominantly containing monocyclic, bicyclic (labdane, halimane, clerodane), and tricyclic (abietane) skeletons. Noteworthy, all these chemical types were separated from semi-polar fractions or semi-polar soluble extracts (acetone, ethyl acetate) utilizing various chromatographic techniques. Labdane diterpenoids (**18**–**70**) with fifty-three derivatives have been characterized as the highest abundance among other phytochemicals. Except for two glycosylated diterpenoids (**69** and **70**), the remaining metabolites of the genus have been identified in aglycosylated form. Recently, phytochemical investigation on the ethanol extract of the fruits of *V. trifolia* yielded four new labdane diterpenoids vitetrolins A-D (**47–48**, **20** and **54**) (Djimabi et al., 2022).

A labdane diterpenoid alkaloid, 9*α*-hydroxy-13 (14)-labden-16,15-amide **68**, with an *α,β*-unsaturated-*γ*-lactam moiety, was isolated from *V. trifolia* leaves (Luo et al., 2017c). Further investigation on the isolation of *V. trifolia* labdane diterpenoid alkaloids led to identification of a cyano-substituted pyrrole cyclic system (**64**–**67**) from the ethanolic extract of the leaf. The precursors of geranylgeranyl pyrophosphate (GGPP), ammonia, and an amino acid may contribute to the biosynthesis of these remarkable metabolites (Luo et al., 2017b).

Halimane diterpenoids (**71**–**81**), known as rearranged labdanes, have further been isolated from this species. According to diterpenoids classification by Roncero and his co-workers, most *V. trifolia* halimane diterpenoids belong to halim-1 (10)-enes (**71**–**73**) and halim-5 (10)-enes (**74**–**80**) groups, described as the largest structures of halimanes (Roncero et al., 2018). The leaves of *V. trifolia* also synthesized three clerodane diterpenoids, named as vitexfolin B (**82**), vitextrifloxide I (**83**), and dysoxydensin G (**84**) (Luo et al., 2017c).

Abietanes diterpenoids (**85**–**89**) have been isolated from the fruit’s extracts or non/semi-polar fractions (Ono et al., 2000; Li et al., 2020b; Djimabi et al., 2021). From this plant, aromatic five abietanes, known as the most abundant naturally occurring abietanes, including abietatrien-3*β*-ol (**86**)**,** ferruginol (**87**), 3*β*-acetoxyabieta-8,11,13-triene-12-ol (**88**), and vitexifolin C (**89**) have been isolated (Gonzalez, 2015).

Djimabi *et al.* identified helipterol (**90**), possessing a monocyclic scaffold, from the fruit alcoholic extract *V. trifolia*, as the only natural source which is so far reported (Djimabi et al., 2021). Moreover, viterotulin D (**37**), vitetrifolin H (**79**), 15,16-epoxy-9-hydroxylabda-13 (16),14-diene (**29**), and 3*β*-acetoxyabieta-8,11,13-trien-12-ol (**88**) were screened out to be the specific secondary metabolites (Wu et al., 2009b). The various class of structures is shown in Figure 2.

#### 5.1.3 Triterpenoids and phytosterols

In *Vitex trifolia*, the most abundant subclasses of triterpenoids identified are oleanane, ursane, and lupane, with **92** to **110** representative compounds (Figure 3). Additionally, one metabolite with a taraxerane skeleton (compound **111**) has been isolated, further enriching the chemical profile of this species (Yong-Sheng Chen et al., 2010; Djimabi et al., 2021). Given the abundance of triterpenoids in their free form in *V. trifolia*, Mohamed et al. have successfully identified three triterpenoid saponins (**97**–**99)** derived from the leaves (Mohamed et al., 2013).

Within the various *Vitex* species, ecdysteroids are of great attention due to their distinctive characteristics advantageous in chemotaxonomy (Sena Filho et al., 2008). Noteworthy ecdysteroids, such as ecdysone (compound **120**), 20-hydroxyecdysone (compound **121**), 20-hydroxyecdysone 2,3-monoacetonide (compound **122**), turkesterone (compound **123**), and polypodine B (compound **124**), have been identified in the leaves of this plant (Thoa et al., 2018). Additionally, this species is known to contain several sterol derivatives—specifically phytosterols (compounds **112–118**) and an ergostanoid (compound **119**)—which have been reported to contribute to the phytochemical diversity of the plant (Huang et al., 2013; Ban et al., 2018; Li et al., 2020b).

### 5.2 Flavonoids

Figure 4 presents a detailed representation of the varied flavonoid subclasses identified within *V. trifolia*, including flavones (entities **125–132**), flavonols (entities **133–142**), and flavanones (entities **143–144**). Among these, a particular focus has been noted on methoxylated flavones, specifically compounds **132** and **135–141**. These compounds are characterized by having between two to five methoxyl groups and have been detected primarily within polar extracts or fractions derived from the fruits and leaves of the plant (Lee et al., 2013; Li et al., 2020a).

Further studies have resulted in the isolation of specific methoxylated flavones, including vitexicarpin (compound **135**), artemetin (compound **138**), and chrysoplenol D (compound **140**) from the seeds (Guan et al., 2010). These isolated compounds have been observed to exist either as free-standing molecules or in glucosidic form, often conjugated mainly with glucose units, as seen in structures **126**, **128–131**, and **136–137**. Furthermore, a distinct glucoside, the neohesperidoside moiety, has been discovered in a conjugated form as quercetin 7-*O*-neohesperidoside (compound **134**), within the ethyl acetate extract of the plant (Mohamed et al., 2013). This array of flavonoid derivations not only highlights the chemical complexity of *V. trifolia* but also signifies the potential of the plant as a resource for multifaceted bioactive compounds.

### 5.3 Lignans, phenylpropanoids and xanthones

Matairesinol 4′-*O*-*β*-D-glucopyranoside **145** was a new lignan isolated from leaves by Ban and his co-workers (Ban et al., 2018). The metabolite **153** dimer structure of dihydro-benzofuran neolignan was separated from the fruit butanol extract (Gu et al., 2008). Recently, six new xanthone dimers (**161–166**) and seven known analogues (**167–173)** have been reported of ethyl acetate extract of *Diaporthe goulteri* L17, *vitex* fungi, phenylpropanoid, and xanthone metabolites of this species are represented in Figure 5 (Peng et al., 2021).

### 5.4 Miscellaneous

Only isolated anthraquinone, physcion **174**, was produced through the polyketide pathway (Chemical metabolites from fruits of *V. trifolia*). Benzoic acid and derivatization (**175–182**) instance vanillin **181** were isolated from different plant parts (Djimabi et al., 2021). Furthermore, saturated, and unsaturated long-chain fatty acids (**185–187**) and alcohol (**188**) were obtained from stems and leaves (Figure 6) (Quan-Yu Liu et al., 2014).

The investigation focused on the impact of four distinct drying methods on the antioxidant properties of *V. trifolia*: microwave-drying, oven-drying, sun-drying, and freeze-drying. The findings indicated that the non-thermal method—freeze-drying—and microwave-drying better preserved the antioxidant properties of the leaves compared to oven-drying and sun-drying, which both resulted in decreased antioxidant properties. In terms of total phenolic content (TPC) and total flavonoid content (TFC), *V. trifolia* leaves retained TPC and TFC values of 4,664 ± 109 GAE/100 g and 637 ± 10 mg QE/100 g, respectively. These values significantly surpass those of mature plants, which showed TPC of 3,229 ± 36 GAE/100 g and TFC of 87.0 ± 0.5 mg QE/100 g) (Chong and Lim, 2012; Chandrasekaran et al., 2019).

### 5.5 Essential oils

Extensive analyses have been performed on essential oils (EOs) from various parts of the *V. trifolia* species, including seeds, leaves, aerial components, and flowers. Notably, an Indian research group achieved the highest yield of EOs from leaves at 5% utilizing steam distillation. Figure 7 illustrates the chemical structures of the major metabolites found within these EOs. *β*-caryophyllene (**202**), a volatile sesquiterpene commonplace across numerous *Vitex* species, has been consistently identified in *V. trifolia*, as referenced in nine previous studies, commanding a majority presence in five of these—specifically within the EOs derived from leaves and flowers (Suksamrarn et al., 1991; Musa et al., 2022). This metabolite has a defensive role in many plants, released in both direct and indirect defence mechanisms against insect and pathogens (Barreto et al., 2021). Moreover, 1,8-cineole (**197**) features prominently in *V. trifolia* essential oils. Variations in the yields and the makeup of EOs metabolites, as presented in Supplementary Table S2, are attributable to such factors as the harvest timing, environmental conditions (including altitude, climate, topography, and soil composition), as well as age and genetic type of the plants (Barra, 2009).

Overall, *V. trifolia* is particularly rich in monoterpene hydrocarbons, ranging from 4.04% to 44.57%, and oxygenated monoterpenes between 6.13% and 31.26%. Metabolites such as *cis*-ocimene (**199**) at 44.57%, *α*-pinene (**191**) ranging from 4.04% to 23.87%, sabinene (**193**) from 9.44% to 39.14%, 1,8-cineole (**197**) at 6.13%–31.26%, and terpinyl acetate (**198**) between 9.6% and 13.48% are among the key chemical constituents characterizing these oils.

## 6 Biological activities of *V. trifolia*

### 6.1 Bioactivities of the extracts

#### 6.1.1 Antioxidant activity

Furthermore, an examination of the antioxidant activities of ethanol extracts from two *Vitex* species, *V. negundo L*., and *V. trifolia* after 30 min exposure to DPPH, revealed that *V. negundo* had greater antioxidant activity ranging from 62.6% to 94.22% with an IC_50_ value between 23.5–208.3 μg/mL. In comparison, *V. trifolia* exhibited a slightly lesser range of antioxidant activity from 60.87% to 89.99% with an IC_50_ value ranging from 40.0 to 226.7 μg/mL. Given the total phenolic and flavonoid content, *V. negundo* showed higher levels with 89.71 mg GAE/g dry weight of the extract and 63.11 mg QE/g dry weight of the extract, respectively, whereas *V. trifolia* had 77.20 mg GAE/g phenolics and 57.41 mg QE/g flavonoids by dry weight of the extract in the *in vitro* study (Saklani et al., 2017).

Additionally, *V. trifolia* demonstrated an IC_50_ value of 64.5 μg/mL in DPPH scavenging activity. This result supports the use of *V. trifolia* as a traditional remedy for ciguatera fish poisoning, endorsing its therapeutic efficacy (Kumar-Roiné et al., 2009).

#### 6.1.2 Hepatoprotective activity

In an *in vivo* study the ethanolic extract of *V. trifolia* flowers at 200 mg/kg b. w. dose exhibited significant hepatoprotective activity against CCl_4_-induced hepatic injury in rats after 7 days treatment, demonstrating remarkable hepatoprotective potential. The observed hepatoprotective effects of the tested metabolite were found to be comparable to those of the standard drug, silymarin, administered at a dosage of 100 mg/kg b. w. with 7 days exposure. This similarity is evident from the significant reduction in the serum levels of key liver enzymes, namely, glutamate pyruvate transaminase (SGPT) (342 U/l in the control group vs. 88 U/l in the treatment group), glutamate oxaloacetate transaminase (SGOT) (358 U/l in the control group vs. 136 U/l in the treatment group), and alkaline phosphatase (ALP) (416 U/l in the control group vs. 180 U/l in the treatment group). Additionally, there is a decrease in the levels of total bilirubin (1.2 mg/dL in the control group vs. 0.8 mg/dL in the treatment group) and gamma-glutamyl transpeptidase (GGTP) (251 U/l in the control group vs. 136 U/l in the treatment group), further confirming the hepatoprotective activity of the metabolite (Anandan et al., 2009). This reduction in enzyme levels underscores the therapeutic value of the treatment in maintaining the liver health.

In an *in vivo* study, the acute toxicity assessments of ethanol and aqueous leaf extracts of *V. trifolia* revealed LD_50_ values of 200 mg/kg b. w., p. o. and 300 mg/kg b. w., p. o., respectively. Subsequently, doses of 20 mg/kg b. w., p. o. and 30 mg/kg, b. w., p. o. of these extracts were selected to evaluate their hepatotoxic activity. The study demonstrated that both extracts, administered for 14 days, significantly reduced the total bilirubin levels. In the context of CCl_4_-induced hepatic toxicity (control group), the total bilirubin levels were 2.50 ± 0.04 mg/dL in the CCl_4_ group compared to 0.63 ± 0.01 and 0.93 ± 0.01 in the CCl_4_ + ethanol extract and CCl_4_ + aqueous extract groups, respectively. Furthermore, the serum marker enzymes showed a decrease in CCl_4_-induced hepatic toxicity, as indicated by the ALT levels decreasing from 1,322.33 IU/L in the CCl_4_ group to 106.43 IU/L and 124.17 IU/L in the CCl_4_ + ethanol extract and CCl_4_ + aqueous extract groups, respectively. Similarly, ALP levels reduced from 442.10 IU/L in the CCl_4_ group to 202.47 IU/L and 236.91 IU/L in the CCl_4_ + ethanol extract and CCl_4_ + aqueous extract groups, respectively. Concomitantly, there was an increase in the total protein levels in the animal subjects experiencing CCl_4_-induced hepatic toxicity, with values of 5.93 ± 0.01 g% in the CCl_4_ group compared to 8.24 ± 0.03 and 8.05 ± 0.03 g% in the CCl_4_ + ethanol extract and CCl_4_ + aqueous extract groups, respectively. These findings suggest the potential hepatoprotective effects of the ethanol and aqueous leaf extracts of *V. trifolia*, as they alleviate CCl_4_-induced hepatic toxicity by modulating key biochemical markers (Manjunatha and Vidya, 2008).

#### 6.1.3 Antispasmodic activity

In an *in vivo* study the assessment of viteosin-A (**34**) and vitexicarpin (**135**), the primary active metabolites present in the n-hexane extract of *V. trifolia*, demonstrated that only vitexicarpin exhibited activity in the tracheospasmolytic bioassay. Notably, this activity was observed at a minimum dose of 1.3 × 10^−5^ M, for 30 min, utilizing sensitized guinea pig trachea stimulated by ovalbumin. The findings suggest that vitexicarpin could potentially hinder the effects of histamine released from sensitized mast cells by stabilizing the membrane function of the mast cells (Alam et al., 2002).

#### 6.1.4 Anti-inflammatory activity

In an *in vivo* study, the anti-inflammatory properties of the hydroalcoholic extract of *V. trifolia* were investigated in rats at doses of 100 mg/kg b. w. and 200 mg/kg b. w. The study demonstrated that the higher dose exhibited an inhibitory effect, as evidenced in the paw volume of 0.37 mL ± 0.01 after 5 h, compared to the control group treated with indomethacin, which had a paw volume of 0.16 mL ± 0.01. The percentage inhibition of inflammation and edema formation at the end of 5 h was 72.23%, while indomethacin demonstrated a higher percentage inhibition of 90.46%. The mean values for total leucocyte count in the group treated with the hydroalcoholic extract were 9,333 ± 448 cells/cm^3^, whereas animals treated with indomethacin showed a count of 11,717 ± 444 cells/cm^3^. In the group treated with 200 mg/kg b. w., the percentage of polymorphonucleocytes and lymphocyte count was 23.33% and 73.67%, respectively, while the indomethacin-treated group showed a count of 23.83% and 70.50%, respectively. Furthermore, the groups treated with the hydroalcoholic extract exhibited reduced levels of macrophages, mast cells, and other inflammatory mediators compared to the control group, indicating its potential to mitigate inflammation (Ankalikar and Viswanathswamy, 2017).

Of the 28 aqueous extracts of plants examined in an *in vitro* study, *V. trifolia* demonstrated a notable ability to inhibit NO production without inducing toxicity. Specifically, at a concentration of 0.25 g/L of the tested extract, aqueous extracts of *V. trifolia* exhibited significant NO inhibitory effects in RAW 264.7 cells that were exposed to 10 μg/mL of lipopolysaccharide (LPS) for 24 h (Kumar-Roiné et al., 2009).

The anti-inflammatory properties of leaf extracts from *V. trifolia* were investigated through animal studies. The results revealed that the extract, administered at 200 mg/kg b. w., exhibited dose-dependent anti-inflammatory effects. At this dosage, both aqueous and ethanolic extracts showed a significant activity (*p* < 0.0001) against the acute inflammatory response, demonstrating reductions of 46.91% and 60.49%, respectively. Although these values were less than that of the reference standard (70.27%) on xylene-induced ear edema, the ethanolic extract inhibited edema notably at the 3rd hour (43%) in carrageenan-induced paw edema compared to other treated groups. This effect was consistent in both carrageenan-induced rat paw edema and xylene-induced ear edema in mice, underscoring the potential of *V. trifolia* as an effective anti-inflammatory agent (Kulkarni, 2011).

An aqueous extract of *V. trifolia* leaf at a concentration of 2,500 μg/mL exhibited an inhibitory activity on the synthesis of interleukin (IL)-1, IL-6, IL-10, and iNOS mRNA with a mild effect on tumor necrosis factor (TNF)-α. These findings indicate that the extract has the potential to modulate the expression of these inflammatory mediators, suggesting its anti-inflammatory properties (Matsui et al., 2009). Furthermore, in an *in vitro* study, it was found that the aqueous leaf extract of *V. trifolia* at a concentration of 5,000 mg/mL and 8 h incubation time displayed a notable inhibitory effect on the expression of multiple inflammatory genes induced by LPS in RAW 264.7 cells. This inhibitory effect was confirmed by a significant decrease in COX-2, CCL-3, and CXCL-10 mRNA production (between 3 and 4-fold compared to the control group). Moreover, the extract exhibited inhibitory activity on LPS-induced p50 mRNA synthesis, further supporting its anti-inflammatory properties (Matsui et al., 2012).

The effect of *V. trifolia* leaf extracts, obtained through different extraction methods, on cytokine production, such as IL-1β and TNF-α in human U937 macrophages, was examined in an *in vitro* study. Among various extraction techniques tested (Soxhlet, ultrasonication, and maceration) and using different solvents, the extracts prepared by maceration in ethanol and ultrasonication in dichloromethane exhibited the highest activity (60%–80% compared to untreated macrophages) in inhibiting IL-1β and TNF-α production in human U937 macrophages after 6 h incubation. These findings suggest that these specific extraction methods and solvents effectively extract bioactive metabolites from *V. trifolia* leaves with potent anti-inflammatory properties in macrophages (Wee et al., 2020).

#### 6.1.5 Anticancer activity and toxicity data

Screening of five samples, namely, *Alpinia galanga* (L.) Willd. (Zingiberaceae), *Piper cubeba* L. f. (Piperaceae), and *Santalum album* L. (Santalaceae), along with *V. trifolia*. at a concentration of 25 μg/mL and incubated for 24 h, revealed a significant inhibitory activity against the T47D breast cancer cell line, with inhibition percentages of 96.4%, 87.6%, 82.6%, and 88.7%, respectively. Epirubicin and doxorubicin were used as positive control, and DMSO for negative control (Dai et al., 2018). The cytotoxic activities of the *V. trifolia* aerial parts were evaluated in an *in vivo* study using three different extracts: methanol, ethyl acetate, and chloroform. The brine shrimp bioassay method was employed for this purpose. The results indicated that the methanolic extract exhibited the highest cytotoxic activity, with an LC_50_ value of 140 mg/mL. The ethyl acetate extract showed slightly lower cytotoxicity, with an LC_50_ value of 165 mg/mL. Lastly, the chloroform extract displayed the least cytotoxicity among the three, with an LC_50_ value of 180 mg/mL, compared with potassium dichromate as the positive control (El-Kousy et al., 2012).

The cytotoxicity of different extracts from *V. trifolia* was assessed on brine shrimp and Hep-G2 cell lines. The tested extracts included the crude hot aqueous-methanol extract, chloroform-methanol extract, ethyl acetate methanol extract, and the residue from the methanol extract. Notably, the residue from the methanol extract exhibited the highest cytotoxic activity, showing an IC_50_ value of 6 μg/mL against Hep-G2 cells. The crude hot aqueous-methanol extract, chloroform-methanol extract, and ethyl acetate methanol extract demonstrated IC_50_ values of 10.7 μg/mL, 20.8 μg/mL, and 65.8 μg/mL, respectively, in comparison to the positive control. These findings indicate varying degrees of cytotoxicity against Hep-G2 cells for different *V. trifolia* extracts, with the residue from the methanol extract displaying the most potent activity. Interestingly, the same trend was observed in the brine shrimp assay (El-Sayed et al., 2011).

In an *in vitro* study, the dichloromethane extracts of *V. trifolia* leaf demonstrated significant toxicity against various cancer cell lines, including SQC-1 UISO, OVCAR-5, HCT-15 COLADCAR, and KB with ED_50_ of 2.2 mg/mL, 2.9 mg/mL, 1 mg/mL, and 1.9 mg/mL, after 72 h incubation respectively. These findings highlight the potential of *V. trifolia* leaf extracts as a source of metabolites with cytotoxic activity against different cancer cell lines (Hernandez et al., 1999).

The synergistic anticarcinogenic effects of ethanolic extracts from *V. trifolia* L. and *Triticum aestivum* L. (Poaceae) were investigated in an *in vitro* study. Both extracts demonstrated anti-degranulation properties individually, and their combination synergistically enhanced the anticarcinogenic potential. This was observed through their ability to inhibit 3,8-diamino-5-ethyl-6-phenylphenanthridinium bromide-induced liver microsomal degranulation. Additionally, these extracts exhibited inhibitory effects on cell proliferation in HCT116 and A549 cell lines. These findings highlight the potential of *V. trifolia* and *T. aestivum* extracts as synergistic agents in cancer prevention and treatment (Mathankumar et al., 2015).

Another *in vitro* study investigated the impact of gamma irradiation treatment at a dose of 7.5 kGy (source: ^60^Co) on the dried coarse powder of legundi leaves, focusing on its potential as an anticancer agent and its chromatogram profile. The IC_50_ values of legundi leaf extract against MCF cancer cells after 72 h of incubation increased from 8.2 μg/mL to 12.1 μg/mL after exposure to the irradiation dose of 7.5 kGy. Similarly, for HeLa cells, the IC_50_ value increased from 7.6 μg/mL to 16.9 μg/mL, and for K-562 cells, it increased from 19.7 μg/mL to 22.4 μg/mL (Winarno et al., 2020).

#### 6.1.6 Anti-amnesic activity

In the passive avoidance and T-maze models, a high dose (20 mg/kg, b. w.) of aqueous *V. trifolia* leaf extract demonstrated a significant (*p* < 0.01) anti-amnesic activity. The extract led to a notably shorter escape latency time (12s) compared to the control group (29s) and showed a maximum percentage of time spent in the probe quadrant by 60.75%. This result was nearly twice as high as that of the control group, indicating improved memory retention compared to both the control and other treatment groups (Mohanbabu et al., 2015).

#### 6.1.7 Larvicidal activity

*Vitex trifolia* demonstrated effective repellency against mosquitoes at a minimal dosage, as evidenced by Gou et al., in 2020 (Gou et al., 2020). Additionally, a study by Tandon et al., in 2008 compared the essential oils (EOs) of both *Vitex trifolia* and *Vitex agnus-castus* L. and found that although the EO of *Vitex agnus-castus* L. was less effective than that of *Vitex trifolia*, both EOs increased larval duration, larval mortality, pupal duration, and adult deformity, while decreasing adult emergence, fecundity, and egg fertility (Tandon et al., 2008).

Chandrasekaran et al. in an *in vivo* study conducted in 2019, revealed that the EO of *Vitex trifolia* exhibited potent larvicidal activity, resulting in 100% mortality against third instar stages of *Aedes aegypti* and *Culex quinquefasciatus* larvae at 125 ppm. The GC-MS analysis of the *Vitex trifolia* EO identified several bioactive metabolites, including eucalyptol (**197**), which accounted for 16.35% of the total peak area, followed by sabinene (**193**) with 9.44%, and *β*-caryophyllene (**202**) with 8.91%, which might contribute to its larvicidal properties (Chandrasekaran et al., 2019). Comparative evaluations of larvicidal efficacy among different Vitex species found that *Vitex trifolia* exhibited the highest larvicidal activity against *C. quinquefasciatus* larvae, with the most effective results. This was also observed in the investigation of the larvicidal activity of fatty acid methyl ester extracts, where *V. trifolia* demonstrated the highest larvicidal activity among the species tested. Furthermore, a comparative study investigated the effects of the extracts from three *Vitex* species on *Anopheles gambiae* s.s. larvae, revealing that the methanol extract of *V. trifolia* leaves and acetone extracts of *V. schiliebenii* stem bark and leaves, as well as *V. payos* (Lour.) Merr. root bark, exhibited significant potency, causing 100% mortality at a concentration of 100 ppm within 72 h. The larvicidal efficacy of four different *Vitex* species was evaluated against *C. quinquefasciatus* larvae, revealing that *V. trifolia* exhibited the highest larvicidal activity with an LC_50_ value of 41.41 ppm (Kannathasan et al., 2007). Among the species examined for their fatty acid methyl ester extracts, *V. trifolia* demonstrated the highest larvicidal activity with an LC_50_ value of 9.25 ppm, highlighting its potent properties (Kannathasan et al., 2008).

Additionally, a comparative *in vivo* study on the effects of extracts from three *Vitex* species on *A. gambiae s.s. larvae* at concentrations ≤ 50 ppm and 24 h time interval, found significant potency in the methanol extract of *V. trifolia* leaves, acetone extracts of *V. schiliebenii* stem bark and leaves, and acetone extract of *V. payos* (Lour.) Notably, *V. schiliebenii* and *V. payos* extracts demonstrated a faster mortality rate in *A. gambiae* s.s. larvae compared to *V. trifolia*, indicating their potential as effective agents for controlling *A. gambiae* s.s. larvae, and their promise for further investigation in mosquito control programs to combat malaria transmission (Nyamoita et al., 2013).

#### 6.1.8 Antimicrobial activity

The *V. trifolia* leaf extracts, tested at a concentration of 200 μg/mL for 30 min incubation time, demonstrated varying degrees of inhibition against different microorganisms in an *in vitro* experiment. The inhibition zone sizes (in mm) for each tested organism were as follows: *Bacillus subtilis*: 15.3 mm; *Staphylococcus aureus*: 14.0 mm; *Pseudomonas aeruginosa*: 13.6 mm; *Proteus mirabilis*: 13.5 mm; *Candida tropicalis*: 12.8 mm; and *Escherichia coli* (*E. coli*): 12.5 mm; *Candida albicans*: 12.0 mm. The zinc oxide nanoparticles (ZnO NPs) coated with an extract of *V. trifolia* exhibited improved MIC value compared to uncoated ZnO NP. The increased antimicrobial activity of the *V. trifolia* leaf extract can be attributed to the presence of vitrifolin A (**59**), the major metabolite in the extract. Vitrifolin A is believed to play a crucial role in enhancing the antimicrobial properties of the extract. It achieves this by binding to the surface of nanoparticles, thus leading to a more effective and targeted delivery of antimicrobial agents. This mechanism enables vitrifolin A to exert a stronger impact on the microorganisms, contributing to the overall enhanced activity of the leaf extract against a range of pathogens (Elumalai et al., 2015).

The *in vivo* antibacterial activity of the ethanolic extract of *V. trifolia* leaves was evaluated at concentrations of 1%, 5%, or 25% against *S. aureus* in the *Drosophila* infection model. The results indicated that 5% and 25% concentrations of the extract exhibited comparable activities. Therefore, the findings suggest that a 5% extract concentration would be sufficient to combat *S. aureus* infection (Sukarsih et al., 2021). Moreover, petrol extract (500 µg/disk) and EtOH extract (400 µg/disk) of *V. trifolia* leaves were moderately active against most of the tested Gram-positive and Gram-negative bacteria except *Klebsiella sp*., *Vibrio choler*a, and *Vibrio mimicus* with a diameter of the zone of inhibition in the range of 8–15 mm (Hossain et al., 2001).

The *in vitro* antibacterial activity of the leaf methanol extracts of *Vitex altissima* L. f, *Vitex diversifolia* Bak., *Vitex negundo* L., *Vitex peduncularis* Wall. ex Schauer and *Vitex trifolia* was examined. *V. peduncularis* showed the highest antimicrobial activity with a zone of inhibition ranging between 11.00 and 22.67 mm; the MIC values were from 62.5 to 1,000.0 μg/mL and the MBC values were from 125.0 to 2000.0 μg/mL (Kannathasan et al., 2011). Methanol, ethanol, and ethyl acetate extract *V. trifolia* were prepared to evaluate the antibacterial activities and showed MIC values of 25, 50, 50, 50, 50, and 25 against *E. coli*, *S. flexneri*, *P. mirabilis*, *P. diminuta*, *E. cloacae,* and *S. aureus* ATCC 6538, respectively (Natheer et al., 2012). Screening of antimicrobial activities on the methanolic extracts of *V. trifolia*, against common freshwater pathogens showed an inhibition zone of 15 mm for *A. hydrophila* and 11 mm for *S. agalactiae*, with no inhibition against *E. cloacae*. Preliminary phytochemical screening of the plant extract showed the presence of tannins, flavonoids, and glycosides (Manaf and Daud, 2016). Antibiofilm screening of *V. trifolia* against *H. pylori* was moderately active (15 mm) with around 60% inhibition at 100 µM (Prasad et al., 2019).

In another *in vitro* study, the antifungal and cytotoxic activities of hexane, methanol, and distilled water extracts of *V. trifolia* were screened against six standard organisms. Results showed that all three extracts were active against *Ceratocystis paradoxa* with MIC in the range of 1.25–5.0 mg/mL, and methanolic and hexanoic extracts showed MIC values of 1.25 and 2.5 mg/mL, respectively. However, these extracts were not potent against *A. niger, P. citrinum*, *M. phaseoli*, and *R. nigricans* (Haripyaree et al., 2021). The hexanoic extract obtained from *V. trifolia* leaves showed remarkable efficacy against the fungal plant pathogen *Fusarium* sp. Within the first 2 days of the experiment, the hexanoic extract completely inhibited the growth of the *Fusarium* sp. However, its inhibitory activity dropped significantly to 15% on day six of the experiment. On the other hand, the dichloromethane extract displayed a significant growth inhibition of 54% against *Fusarium* sp. within 4 days of the experiment (Hernandez et al., 1999).

#### 6.1.9 Antiviral activity

*V.* *trifolia* demonstrated significant antiviral activity against *Molluscum contagiosum* and *Herpes simplex*, with effective concentrations of approximately 0.25 μg/mL and 0.5 μg/mL, respectively, at a 0.4 μg/mL concentration in an *in vitro* assay. Importantly, this antiviral efficacy was achieved without causing notable toxicity. These findings highlight the potential of *V. trifolia* as a promising natural source for developing safe and effective antiviral agents. Further exploration into the specific bioactive metabolites and their mechanisms of action, as well as broader applications in clinical settings, would enhance our understanding of the therapeutic potential of *V. trifolia* in antiviral interventions (Vimalanathan et al., 2009).

#### 6.1.10 Anti-HIV activity

In a research study, the impact of aqueous and 80% ethanol extracts from 20 medicinal plants of Thai on HIV type 1 reverse transcriptase activity was investigated. The results revealed that the water extracts of *Vitex glabrata* R. Br. (branch), *V. trifolia*. (aerial part), and *Vitex negundo* L. (aerial part) displayed a remarkably good inhibition ratio (% IR) higher than 90% at a concentration of 200 μg/mL in 1 h incubation. Doxorubicin hydrochloride, as a positive control, inhibited the HIV-1 RT activity at 1 mM by 98.3%. These findings suggest that these specific extracts from *V. glabrata*, *V. trifolia*, and *V. negundo* possess a strong potential as candidates for further investigation in the development of anti-HIV therapies due to their significant inhibitory effects on HIV-1 reverse transcriptase activity (Woradulayapinij et al., 2005).

#### 6.1.11 Anti-malaria activity

In an investigation utilizing semi-structured questionnaires and informant interviews to gather knowledge about plants associated with malaria and related symptoms, the antimalarial potential of the extracts from 70 plant species, representing 62 genera and 34 families, was evaluated. The results highlighted Solanaceae as the most frequently cited family, with 7 species showing promising antimalarial properties. Noteworthy results were observed within the Lamiaceae family, specifically *Vitex negundo* L. and *Vitex trifolia* L., identified as antimalarial agents, with documentation from the Soon Valley region in Pakistan for the treatment of malaria. These findings underscore the ethnobotanical importance of certain plant species within communities for their potential antimalarial properties (Shah and Rahim, 2017).

#### 6.1.12 Respiratory disorder

In an *in vitro* study screening the inhibitory effect of alcoholic and hexanoic extracts of *V. trifolia* on histamine release from RBL-2H3 cells revealed that 0.5 mg/mL resulted in more than 80% inhibition of IgE-dependent histamine release from RBL-2H3 cells (Ikawati et al., 2001). A separate study demonstrated that combining *Curcuma xanthorrhiza* Roxb. rhizome (Zingiberaceae; *Curcumae xanthorrhizae* rhizoma), *V. trifolia* leaves, *Zingiber officinale* Roscoe. rhizome (Zingiberaceae; *Zingiberis* rhizoma) and *Echinacea purpurea* (L.) Moench herb (Asteraceae) exhibited synergistic immunomodulatory effects. The combination, administered at 490 mg/kg and 980 mg/kg, significantly enhanced the macrophage phagocytic index, reaching 15.29 µgr/mL and 26.78 µgr/mL, respectively. These values were compared to *E. purpurea* as a positive control, with a phagocytic index of 12.53 ± 1 µgr/mL at 750 mg. Moreover, the combination increased the production of IgG antibodies, with concentrations reaching 35 µgr/mL and 38 µgr/mL at doses of 490 mg/kg and 980 mg/kg, respectively. Again, these values were compared to *E. purpurea* as a positive control, which showed a 35 µgr/mL concentration at 750 mg. This study highlights the potential synergistic immunomodulatory effects of combining these botanical drugs (Ikawati et al., 2019).

#### 6.1.13 Wound healing effect

In a comparative analysis of wound healing potential, the ethanol leaf extract of *V. trifolia* demonstrated superior activity compared to *Vitex altissima* L. f*.*. The incision wound tissue tensile strength for the positive control was 600.00, while it was 578.20 for *V. trifolia* and 529.08 for *V. altissima*. Hydroxyproline levels, indicative of collagen formation, were higher in the ethanol leaf extract of *V. trifolia* (2,567 µg/100 mg) compared to *V. altissima* (2012 µg/100 mg), with a negative control registering at 1943 µg/100 mg. Granuloma dry weight, a measure of tissue healing, was notably higher in *V. trifolia* (157.30 mg/100 g) compared to *V. altissima* (136.50 mg/100 g), with the control at 33 mg/100 g. Additionally, in dead space wound tissue tensile strength, *V. trifolia* demonstrated enhanced strength (491.20 g) compared to *V. altissima* (430.50 g), surpassing the positive control (181 g). These findings indicate that *V. trifolia* significantly improves the quality of wound healing and scar formation, outperforming *V. altissima* in various wound healing parameters (Manjunatha et al., 2007).

### 6.2 Bioactivities of pure metabolites

Labdane-type diterpenes isolated from *V. trifolia*, namely, vitexilactone (**30**), (5S,6R,8R,9R, 10S)-6-acetoxy-9-hydroxy-13 (14)-labden-16,15-olide (**39**), rotundifuran (**26**), vitetrifolin D (**76**), and vitetrifolin E (**77**), exhibited remarkable effects on cellular processes. In an *in vitro* study, at higher concentrations (100.0 μg/mL), these metabolites demonstrated a significant induction of apoptosis in both tsFT210 and K562 cells. Conversely, at lower concentrations, they impeded the cell cycle progression of both tsFT210 and K562 cells, specifically at the G0/G1 phase (Li et al., 2005b).

Six flavonoids, persicogenin (**143**), artemetin (**138**), luteolin (**127**), penduletin (**141**), vitexicarpin (**135**) and chrysosplenol-D (**140**), were isolated from *V. trifolia* inhibited the proliferation of sFT210 cancer cells with the IC_50_s > 100 μg/mL (inhibition rate at 100 mg/mL was 21,47.9%) for persicogenin > 100 μg/mL (inhibition rate at 100 mg/mL was 49.6%) for artemetin, 10.7 μg/mL for luteolin, 19.8 μg/mL for penduletin, 0.3 μg/mL for vitexicarpin, and 3.5 μg/mL for chrysosplenol-D in 17 h treatment. It was shown that the mentioned metabolites exerted their anti-proliferative effect on tsFT210 cells *via* inhibiting the cell cycle and inducing apoptosis (Li et al., 2005c).

A variety of diterpenoids, including vitetrifolin I, D, E, F, H, (**75–79**), vitexoid (**15**), as well as vitexilactone (**30**), 6-acetoxy-9-hydroxy-13 (14)-labdane-16,15-olide (**39**), previtexilactone (**41**), and 6-acetoxy-9,13; 15,16-diepoxy-15-methoxylabdane (**53**), were isolated from the fruits of *V. trifolia*. These metabolites demonstrated inhibitory effects on the proliferation of Hela cells, with IC_50_ values ranging from 4 to 28 μM. Vitetrifolin I exhibited the strongest potency, inducing cell cycle arrest at the G0/G1 phase and promoting apoptosis in Hela cells (Wu et al., 2009a). On the other hand, seven labdane-type diterpenoids, namely, vitextrifolins A−G (**18–19**, **21–25**), derived from the fruits of *V. trifolia*, did not exhibit significant toxicity (IC_50_ < 5 μg/mL) against various cell lines, including human colon carcinoma (HCT116) in an *in vitro* study, human lung adenocarcinoma (A-549), human promyelocytic leukaemia (HL-60), and human breast carcinoma (ZR-75–30) (Zheng et al., 2013a). Twenty-seven diterpenoids derived from *V. trifolia* were examined for their inhibitory activity against DNA topoisomerase I. Among these metabolites, vitextrifloxide G (**72**) and vitextrifloxide I (**83**) demonstrated remarkable potency by exhibiting more than 81% inhibition at a concentration of 100 µM. To further assess the effectiveness of vitextrifloxide G, Luo et al.evaluated it using the MTT method in human colorectal carcinoma cells (HCT116), resulting in an IC_50_ value of 20.3 µM (Luo et al., 2017c).

Diaporxanthone A (**161**) and diaporxanthone F (**166**) displayed notable antifungal properties against *Nectria sp*. and *C. musae* (ACCC 31244), in the *in vitro* study. Diaporxanthone A exhibited antifungal activity at a minimum dosage of 10.0 μg/scrip, while diaporxanthone F demonstrated effectiveness at a lower dosage of 2.5 μg/scrip (Peng et al., 2021).

*In vitro* minimum lethal concentrations of the isolated metabolites of *V. trifolia* against epimastigotes of *Trypanosoma cruzi* were 11 mM for 9,13-epoxy-16-norlabda-13*E*-en-15-aL (**61**), 36 mM for 6*β*-acetoxy-9*α*,13-epoxy-16-norlabd-13*E*-en-15-aL (**63**), 34 for mM vitexifolin E (**60**), 34 mM of vitexifolin F (**78**), 66 mM of vitexilactone (**30**), 66 mM of (6-acetoxy-9-hydroxy-13 (14)-labden-16,15-olide) (**39**), and 265 mM of previtexilactone (**41**) (Kiuchi et al., 2004). Besides the bioactivities of the isolated metabolites from *V. trifolia*, the following metabolites can be highlighted as the major known ones.

#### 6.2.1 Casticin

Casticin (**135**), a flavonoid isolated from *V. trifolia*, has demonstrated anti-inflammatory and antitumor effects on ADTC5 cells. However, it exhibited significant toxicity at a concentration of 40 μM after 2 h of treatment. In a dose-dependent manner at concentrations of 10, 20, and 30 μM, in 2 h incubation time, casticin reduced proinflammatory cytokines such as IL-6, TNF-α, and prostaglandin E2 (PGE2), as well as oxidative stress markers including MDA and inducible nitric oxide synthase (iNOS) expression. Specifically, at 30 μM, casticin downregulated IL-6 protein expression in IL-1β-stimulated ADTC5 to 40 pgr/mL compared to 67 pgr/mL in the control group. It also downregulated TNF-α protein expression to 50 pgr/mL from 110 pgr/mL in the control group and reduced PGE2 protein expression to 600 pgr/mL from 1,150 pgr/mL in the control group. These findings highlight the potential of casticin in modulating inflammatory responses and oxidative stress (Chu et al., 2020).

In a murine asthma model, administering casticin at doses of 5 or 10 mg/kg b. w. effectively mitigated airway hyperresponsiveness, airway inflammation, and oxidative stress in the lungs. These beneficial effects were attributed to regulating Th2 cytokine and chemokine gene expression within the lung. Casticin also demonstrated significant suppression of proinflammatory cytokine levels and eotaxin. Specifically, casticin reduced the IL-6 levels compared to the ovalbumin (OVA)-induced asthma group (70.4 ± 4.4 pg/mL in the control group vs. 37.4 ± 4.1 pg/mL in the prednisolone positive control; casticin at doses of 5: 57.7 ± 5.9 pg/mL, and casticin at doses of 10: 42.1 ± 7.1 pg/mL). Furthermore, casticin increased the INF-γ levels compared to the OVA group (93.6 ± 11.7 pg/mL in the control group vs 49.6 ± 9.5 pg/mL in the prednisolone positive control; casticin at doses of 5: 109.5 ± 9.5 pg/mL, and casticin at doses of 10: 121.9 ± 23.7 pg/mL). Additionally, it successfully reduced the adherence of THP-1 monocyte cells to BEAS-2B cells by suppressing ICAM-1 expression, with ICAM-1 level decreasing from 1,300 pgr/mL in control to approximately 600 pgr/mL with 20 µM casticin. These findings underscore the potential of casticin as a therapeutic intervention for asthma-related inflammation (Liou et al., 2018).

#### 6.2.2 Rotundifuran

Rotundifuran (**26**) demonstrates notable suppression of cervical cancer cell lines, particularly HeLa and SiHa cells, with an IC_50_ of less than 10 μM in 24 and 48 h treatments, indicating its potent anti-proliferative activity. This suppression is attributed to the induction of apoptosis *in vitro*, underscoring its potential as an effective antitumor agent. Antitumor properties of rotundifuranare associated with its capability to target ROS-induced mitochondrial-dependent apoptosis involving the MAPK and PI3K/Akt signalling pathways. *In vivo* studies further validate the antitumor effects of rotundifuran, showcasing a reduction in tumor size to around 190 mm^3^ at 40 mg/kg, in comparison to cis-platinum treatment as a positive control at 3 mg/kg/3 days, resulting in tumor size of 200 mm^3^. These findings highlight the promising potential of rotundifuran as a therapeutic agent against cervical cancer (Gong et al., 2021).

#### 6.2.3 Artemetin

In an *in vitro* study, artemetin (**138**) demonstrated suppressive effects on TNF-α and IL-1β at concentrations of 50 μg/mL and 100 μg/mL, leading to a reduction of TNF-α levels to 20% and IL-1β levels to 30% of their original concentrations after 48 h treatment. Dexamethasone at 64.4 ng/mL was the positive control, exhibiting a 0.25 and 40-fold change, respectively. Moreover, after 48 h of incubation, artemetin displayed inhibitory effects on the cell growth in U937 macrophages, with IC_50_ values of 125.6 ± 15.3 μg/mL (323.4 ± 39.3 μM). These results suggest the potential of artemetin in modulating inflammatory responses and inhibiting the growth of U937 macrophages (Wee et al., 2020).

#### 6.2.4 Methyl-p-hydroxybenzoate

In an *in vivo* study, the larvicidal activities of methyl-*p*-hydroxybenzoate (**182**), isolated from the methanol extract of *V. trifolia* leaves, were evaluated against early 4^th^ instar larvae of *C. quinquefasciatus* and *A. aegypti* mosquitoes. Remarkably, the metabolite exhibited complete mortality of the larvae for both mosquito species at a concentration of 20 ppm. The LC_50_ values were 5.77 ppm for *C. quinquefasciatus* and 4.74 ppm for *A. aegypti*. These findings underscore the potent larvicidal activity of methyl-phydroxybenzoate against the larvae of these disease-carrying mosquito species, suggesting its potential application as an effective and eco-friendly agent in mosquito control programs (Kannathasan et al., 2011).

#### 6.2.5 Vitepyrroloid A

The metabolite vitepyrroloid A (**64**), when evaluated on the human nasopharyngeal carcinoma cell line CNE1, exhibited cytotoxic activity with an IC_50_ value of 8.7 μM after 3 days. This is compared to cisplatin as positive control, which had an IC_50_ value of 4.6 ± 0.1 μM. These findings suggest the potential cytotoxic efficacy of the metabolite against CNE1 cells, although cisplatin demonstrated a slightly lower IC_50_ value in this context (Luo et al., 2017c).

#### 6.2.6 Vitextrifloxide G

Vitextrifloxide G (**72**) exhibited an IC_50_ value of 20.3 µM against HCT 116 human colorectal carcinoma cells in an *in vitro* study (Luo et al., 2017c).

#### 6.2.7 Vitexilactone

In an *in vitro* experiment, vitexilactone (**30**) demonstrated the ability to enhance lipid accumulation and promote the expression of adiponectin and GLUT4 on the membrane of 3T3-L1 cells. Moreover, it effectively reduced the size of adipocytes and suppressed the phosphorylation of IRS-1, ERK1/2, and JNK in 3T3-L1 cells through PPARγ mediation. These findings suggest that vitexilactone holds promise as a potential candidate for developing improved antidiabetic agents (Nishina et al., 2017).

#### 6.2.8 (-)-O-Methylcubebin

In an *in vitro* study, (-)-*O*-methylcubebin (**149**) exhibited significant antidiabetic properties at doses ranging from 1.5 to 50 µM. (-)-*O*-Methylcubebin showed no toxicity at 50 µM or less after 48 h of incubation in 3T3-L1 cells. It reduced the size of the adipocyte and facilitated the expression of proteins associated with adipogenesis, including adiponectin. Molecular analysis revealed that methylcubebin acted as an agonist for PPARγ, thereby promoting adipogenesis by inhibiting the phosphorylation of extracellular signal-regulated kinase 1/2 (ERK1/2) and p38MAPK (Ukiya et al., 2019a).

#### 6.2.9 Vitexicarpin

Vitexicarpin (**135**) exhibited substantial inhibitory effects on the growth of human cancer cells. In *an in vitro* study, it displayed IC_50_ values of 0.44 µM and 0.28 µM against HT-1080 and K562 cells, respectively, after 24 h and IC_50_ values of 19 μM and 0.66 µM against A2780 and HCT-15 cells after 48 h of treatment. This metabolite also led to distinct morphological changes indicative of apoptosis, and flow cytometric analysis revealed a dose-dependent sub-G0/G1 peak (Wang et al., 2005).

#### 6.2.10 9-Hydroxy-13 (14)-labden-15,16-olide

9-hydroxy-13 (14)-labden-15,16-olide (**36**) displayed noteworthy efficacy against *Mycobacterium tuberculosis* H37Rv, with a MIC of 100 μg/mL in an *in vitro* study, compared to streptomycin, a reference antibiotic, exhibited a lower MIC value of 2.0 μg/mL. These results highlight the potential antimycobacterial activity of 9-hydroxy-13 (14)-labden-15,16-olide, despite its MIC being higher than the reference streptomycin (Tiwari et al., 2013).

#### 6.2.11 Isoambreinolide

In the BACTEC-460 assay, isoambreinolide (**55**) displayed notable antimycobacterial properties, demonstrating significant antitubercular activity against *M. tuberculosis* H37Rv. The MIC of isoambreinolide was determined to be 25 μg/mL, while streptomycin, a reference antibiotic, showed a lower MIC value of 2.0 μg/mL. These results indicate the potential of isoambreinolide as an antimycobacterial agent, albeit with a slightly higher MIC than the reference (Tiwari et al., 2013).

#### 6.2.12 Diaporxanthone D

In an *in vitro* experiment, diaporxanthone D (**164**) exhibited notable cytotoxic effects on EC109, A2870, HepG2, PC3, A549, and HBE cell lines, with IC_50_ values of 1.88, 8.11, 4.49, 1.66, 8.4 and 6.5 μM, respectively (Peng et al., 2021).

#### 6.2.13 Miscellaneous

In an *in vitro* study, Bao et al. evaluated diterpenoid glucoside, (3S,5S,6S,8R,9R, 10S)-3,6,9-trihydroxy-13 (14)-labdean-16,15-olide 3-*O*-*β*-D-glucopyranoside (**69**), and an iridoid glucoside, (1S, 5S,6R, 9R)-10-*O*-*p*-hydroxybenzoyl-5,6*β*-dihydroxy iridoid 1-*O*-*β*-D-glucopyranoside (**3**) along with viteagnuside A (**70**), 10-*O*-vanilloylaucubin (**2**), agnusoside (**6**), nishindaside (**7**), 3-normal-butyl-nishindaside (**8**) and 3-normal-butyl-isonishindaside (**9**) isolated from *V. trifolia* on nitric oxide production in LPS-induced RAW 264.7 macrophages. Among the tested metabolites, (1S,5S,6R, 9R)-10-*O*-*p*-hydroxybenzoyl-5,6*β*-dihydroxy iridoid 1-*O*-*β*-D-glucopyranoside, 10-*O*-vanilloylaucubin, agnusoside, and 3-normal-butyl-nishindaside displayed moderate inhibitory activities, with IC_50_ values of 90.05 μM, 88.51 μM, 87.26 μM, and 76.06 μM, respectively. These values were compared to hydrocortisone as a positive control, which exhibited an IC_50_ value of 58.79 ± 3.32 μM (Bao et al., 2018).

## 7 Therapeutic development goals

Utilizing the identified bioactive metabolites, the initiation of a new phase of research focuses on the targeted design and experimental testing of pharmaceutical interventions. With this strategic approach, the intention is to position *V. trifolia* and its bioactive metabolites as potential sources for the development of effective treatments across a broader spectrum of diseases, including neurodegenerative disorders, cardiovascular diseases, metabolic disorders, and infectious diseases. This potential is attributed to their high concentrations of antioxidants, phenolic metabolites, and flavonoids. By broadening the focus beyond the initial therapeutic targets, efforts will be made to uncover new possibilities and contribute to the growing body of knowledge on the versatile pharmacological effects of *V. trifolia*.

Beyond the traditional approach to drug development, cutting-edge methodologies are incorporated to harness nanotechnology for enhanced drug delivery and improved bioavailability. The feasibility of nanoformulations derived from *V. trifolia* should be investigated, exploring their potential in targeted drug delivery systems designed to enhance efficacy, reduce side effects, and facilitate the penetration of bioactive metabolites into specific cells or tissues.

Finally, the intention was to present a roadmap for future research directions, emphasizing the translation of laboratory findings into clinical applications and the continued investigations into the therapeutic potential of *V. trifolia* and its bioactive metabolites. This comprehensive approach aims to bridge the gap between traditional knowledge and practical medical applications, with the boundaries of *V. trifolia* research intended to improve healthcare.

## 8 Conclusion

This comprehensive review has documented the intricate phytochemical profile of *V. trifolia*, particularly emphasizing its diverse ethnomedicinal applications. The identification of a myriad of bioactive compounds, encompassing a broad spectrum of terpenoids, flavonoids, lignans, phytosterols, phenylpropanoids, and xanthones, with a notable abundance of labdane diterpenoids, adds depth to our understanding.

Furthermore, the elucidation of the biosynthetic pathways for terpenoids, flavonoids, and iridoids provides a crucial foundation for exploring the molecular mechanisms underlying the synthesis of these bioactive constituents in *V. trifolia*.

Modern pharmacological investigations affirm the extensive therapeutic role of *V. trifolia* in addressing diverse health issues, ranging from tendon-and-bone-related conditions to infections and inflammations. Notably, the research underscores the potent biological activities of the plant, highlighting its antioxidant, anti-inflammatory, hepatoprotective, and anti-cancer properties. The confirmed antimicrobial, antiviral, anti-malarial, and anti-spasmodic effects further underscore the medicinal efficacy inherent in *V. trifolia*.

Moreover, advancements in analytical techniques offer deeper insights into the phytochemistry of *V. trifolia*, paving the way for identifying and characterizing novel secondary metabolites. Integrating highly precise analytical methods with bioassay-guided fractionation enriches our understanding of the plant’s phytochemistry and establishes a framework for future pharmacological explorations. This review, thus, contributes to the evolving landscape of *V. trifolia* research, providing a platform for continued investigation into its therapeutic potential.

Furthermore, due to the lack of clinical trials, our study emphasizes the necessity to conduct such trials to test the plant products’ efficacy. However, it is essential to highlight that further toxicological investigations are required to explore safety aspects comprehensively. Moreover, investigating the bioavailability of the plant’s bioactive chemicals provides valuable insights into their absorption and distribution, making a substantial contribution to a more profound understanding of their potential applications.

The investigation of medicinal plants, including *V. trifolia*, encounters challenges and limitations such as variability in bioactive compounds, complex synergistic effects, and a lack of standardization; asides from limited clinical evidence, potential adverse effects, regulatory issues, and ethical concerns in harvesting further constrain research. Our study proposes conducting rigorous scientific investigations and fostering collaboration to bridge the gap between traditional and modern methodologies, using *V. trifolia* as a focal point.

## 9 Perspectives

The exploration of the compounds within *V. trifolia* is recommended, with a specific focus on integrating advanced bioinformatics tools, particularly machine learning algorithms, and simulated modelling applications such as Swiss ADME, Pkcsm, and Qikprop. The objective is to anticipate the potential of predicting the interactions and characteristics of the bioactive compounds present in *V. trifolia*. The application of predictive modelling holds promise in the pathway from initial compound discovery to therapeutic application. Moreover, recognizing the imperative need for translational research, the call for clinical trials within this category is underscored. Including clinical trials is anticipated to bridge the gap between experimental findings and real-world therapeutic applications. This crucial step validates the efficacy of *V. trifolia*-derived compounds and generates essential data for potential future pharmaceutical advancements. Integrating bioinformatics and clinical trials will synergistically propel *V. trifolia* research into a transformative phase, offering novel therapeutic possibilities for global health improvement.

## Abbreviations

*V. trifolia* L., *V. trifolia*; GGPP, Geranylgeranyl pyrophosphate; EOs, Essential oils; TPC, Total phenolic content; TFC, Total flavonoid content; SGPT, Glutamate pyruvate transaminase; SGOT, Glutamate oxaloacetate transaminase; ALP, Alkaline phosphatase; NO, Nitric oxide; LPS, Lipopolysaccharide; IL, Interleukin; TNF-α, Tumor necrosis factor; ZnO NPs, Zinc oxide nanoparticles; MCV, *M. contagiosum* virus; HSV, Herpes simplex virus; PGE2, Prostaglandin E2; ERK1/2, Extracellular signal-regulated kinase 1/2; LT_50_, Lethal time to cause 50% mortality; MTT assay, 3-(4,5-dimethylthiazol-2-yl)-2,5-diphenyl-2H-tetrazolium bromide assay.

## References

[B1] Adiyasa I. W. S. Santi S. R. Manurung M. (2014). Uji Aktivitas repelan minyak atsiri buah liligundi (*Vitex trifolia* Linn) terhadap nyamuk *Aedes aegypti* . J. Kim. 8, 23–27.

[B2] Alam G. Wahyuono S. Ganjar I. G. Hakim L. Timmerman H. Verpoorte R. (2002). Tracheospasmolytic activity of viteosin-A and vitexicarpin isolated from *Vitex trifolia* . Planta Med. 68, 1047–1049. 10.1055/s-2002-35650 12451502

[B3] Alyasin S. Nabavizadeh S. H. Esmaeilzadeh H. Heydari S. T. Mosavat S. H. Parvizi M. M. (2020). Efficacy of oral supplementation of whey protein in patients with contact dermatitis: a pilot randomized double-blind placebo-controlled clinical trial. Dermatol. Ther. 33, e14260. 10.1111/dth.14260 32876987

[B4] Anandan R. Jayakar B. Karar B. Babuji S. Manavalan R. Kumar R. S. (2009). Effect of ethanol extract of flowers of *Vitex trifolia* Linn. on CCL4 induced hepatic injury in rats. Pak J. Pharm. Sci. 22, 391–394. 19783517

[B5] Ankalikar A. Viswanathswamy A. H. (2017). Effect of leaves of *Vitex trifolia* Linn on different stages of inflammation. Indian J. Pharm. Educ. 51, 461–471. 10.5530/ijper.51.3.74

[B6] Arpiwi N. L. Muksin I. K. Kriswiyanti E. (2020). Essential oils from *Vitex trifolia* as an effective repellent for *Aedes aegypti* . Biodiversitas J. Biol. Divers 21. 10.13057/biodiv/d211060

[B7] Ayeni E. A. Gong Y. Yuan H. Hu Y. Bai X. Liao X. (2022). Medicinal plants for anti-neurodegenerative diseases in West Africa. J. Ethnopharmacol. 285, 114468. 10.1016/j.jep.2021.114468 34390796

[B8] Ban N. K. Thoa N. T. K. Linh T. M. Giang V. H. Trang D. T. Nhiem N. X. (2018). Chemical constituents of *Vitex trifolia* leaves. Nat. Prod. Commun. 13, 1934578X1801300. 10.1177/1934578x1801300205

[B9] Ban Y. Xia T. Jing R. Guo Y. Geng Y. Ye Q. (2020). Vitex diterpenoids: structural diversity and pharmacological activity. Curr. Pharm. Des. 26, 138–159. 10.2174/1381612825666191216151703 31840598

[B10] Bao F. Tang R. Cheng L. Zhang C. Qiu C. Yuan T. (2018). Terpenoids from *Vitex trifolia* and their anti-inflammatory activities. J. Nat. Med. 72, 570–575. 10.1007/s11418-018-1178-x 29429059

[B11] Barra A. (2009). Factors affecting chemical variability of essential oils: a review of recent developments. Nat. Prod. Commun. 4, 1934578X0900400–1154. 10.1177/1934578x0900400827 19769002

[B12] Barreto I. C. De Almeida A. S. Sena Filho J. G. (2021). Taxonomic insights and its type cyclization correlation of volatile sesquiterpenes in vitex species and potential source insecticidal compounds: a review. Molecules 26, 6405. 10.3390/molecules26216405 34770814 PMC8587464

[B13] Blois M. S. (1958). Antioxidant determinations by the use of a stable free radical. Nature 181, 1199–1200. 10.1038/1811199a0

[B14] Chan E. W. C. Baba S. Chan H. T. Kainuma M. Tangah J. (2016). Medicinal plants of sandy shores: a short review on *Vitex trifolia* L. and Ipomoea pes-caprae (L.) R. Br. Indian J. Nat. Prod. 7, 107–115.

[B15] Chandrasekaran T. Thyagarajan A. Santhakumari P. G. Pillai A. K. B. Krishnan U. M. (2019). Larvicidal activity of essential oil from *Vitex negundo* and *Vitex trifolia* on dengue vector mosquito *Aedes aegypti* . Rev. Soc. Bras. Med. Trop. 52, e20180459. 10.1590/0037-8682-0459-2018 31365621

[B16] Chen Y.-S. Xie J.-M. Yao H. Lin X.-Y. Zhang Y.-H. (2010). Studies on the triterpenoids of *Vitex trifolia* . J. Chin. Med. Mater 33, 908–910. 21049612

[B17] Cheng A.-X. Lou Y.-G. Mao Y.-B. Lu S. Wang L.-J. Chen X.-Y. (2007). Plant terpenoids: biosynthesis and ecological functions. J. Integr. Plant Biol. 49, 179–186. 10.1111/j.1744-7909.2007.00395.x

[B18] Chong K. Lim Y. Y. (2012). Effects of drying on the antioxidant properties of herbal tea from selected Vitex species. J. Food Qual. 35, 51–59. 10.1111/j.1745-4557.2011.00422.x

[B19] Chu J. Yan B. Zhang J. Peng L. Ao X. Zheng Z. (2020). Casticin attenuates osteoarthritis-related cartilage degeneration by inhibiting the ROS-mediated NF-κB signaling pathway *in vitro* and *in vivo* . Inflammation 43, 810–820. 10.1007/s10753-019-01167-y 31897918

[B20] Conti M. V. Guzzetti L. Panzeri D. De Giuseppe R. Coccetti P. Labra M. (2021). Bioactive compounds in legumes: implications for sustainable nutrition and health in the elderly population. Trends Food Sci. Technol. 117, 139–147. 10.1016/j.tifs.2021.02.072

[B21] Dai M. Fadhilah A. Rahmawati J. Forentin A. Usia T. Maryati M. (2018). T47D cell-inhibiting Indonesian medicinal plants and active constituents of *Alpinia galanga* rhizome. Pharmacogn. Mag. 14, 359–363. 10.4103/pm.pm_259_17

[B22] Das R. Mitra S. Tareq A. M. Emran T. B. Hossain M. J. Alqahtani A. M. (2022). Medicinal plants used against hepatic disorders in Bangladesh: a comprehensive review. J. Ethnopharmacol. 282, 114588. 10.1016/j.jep.2021.114588 34480997

[B23] De Kok R. P. (2007). The genus *Vitex* L.(Lamiaceae) in New Guinea and the south pacific islands. Kew Bull., 587–603.

[B24] Devi W. R. Singh C. B. (2014). Chemical composition, anti-dermatophytic activity, antioxidant and total phenolic content within the leaves essential oil of *Vitex trifolia* . Int. J. Phytocosmet Nat. Ingred. 1, 5. 10.15171/ijpni.2014.05

[B25] Dewick P. M. (2002). Medicinal natural products: a biosynthetic approach. Hoboken, New Jersey, United States: John Wiley and Sons. 10.1002/9780470742761

[B26] Dhanani T. Shah S. Kumar S. (2015). A validated high performance liquid chromatography method for determination of three bioactive compounds p-hydroxy benzoic acid, negundoside and agnuside in Vitex species. Maced. J. Chem. Chem. Eng. 34, 321–331. 10.20450/mjcce.2015.500

[B27] Djimabi K. Li B. Chen X.-H. Su P.-J. Liu X. Wang R.-Y. (2021). Chemical constituents from the fruits of *Vitex trifolia* L. (Verbenaceae) and their chemotaxonomic significance. Biochem. Syst. Ecol. 97, 104305. 10.1016/j.bse.2021.104305

[B28] Djimabi K. Wang R.-Y. Li B. Chen X.-H. Liu X. Wang M.-J. (2022). Diterpenoids with α-glucosidase inhibitory activities from the fruits of *Vitex trifolia* Linn. Fitoterapia 161, 105248. 10.1016/j.fitote.2022.105248 35777590

[B29] El-Kousy S. Mohamed M. Mohamed S. (2012). Phenolic and biological activities of *Vitex trifolia* aerials parts. Life Sci. J. 9, 670–677.

[B30] El-Sayed M. M. El-Hashash M. M. Mohamed M. A. Korany T. M. (2011). Cytotoxic activity of *Vitex trifolia* purpurea extracts. J. Egypt Soc. Parasitol. 41, 409–416. 21980779

[B31] Elumalai K. Velmurugan S. Ravi S. Kathiravan V. Raj G. A. (2015). Bio-approach: plant mediated synthesis of ZnO nanoparticles and their catalytic reduction of methylene blue and antimicrobial activity. Adv. Powder Technol. 26, 1639–1651. 10.1016/j.apt.2015.09.008

[B32] Fang S.-M. Liu R. Li L. Yao J.-L. Liu E.-W. Fan G.-W. (2019). Anti-inflammatory diterpenes from the fruits of *Vitex trifolia* L. var. simplicifolia Cham. J. Asian Nat. Prod. Res. 21, 985–991. 10.1080/10286020.2018.1482881 29996686

[B33] Gong G. Shen Y.-L. Lan H.-Y. Jin J.-M. An P. Zhang L.-J. (2021). The Cyr61 is a potential target for rotundifuran, a natural labdane-type diterpene from *Vitex trifolia* L., to trigger apoptosis of cervical cancer cells. Oxid. Med. Cell. Longev. 2021, 6677687. 10.1155/2021/6677687 34234887 PMC8218918

[B34] Gonzalez M. A. (2015). Aromatic abietane diterpenoids: their biological activity and synthesis. Nat. Prod. Rep. 32, 684–704. 10.1039/c4np00110a 25643290

[B35] Gou Y. Li Z. Fan R. Guo C. Wang L. Sun H. (2020). Ethnobotanical survey and evaluation of traditional mosquito repellent plants of Dai people in Xishuangbanna, Yunnan Province, China. J. Ethnopharmacol. 262, 113124. 10.1016/j.jep.2020.113124 32730874

[B36] Guan R. Wang D. Yu Z. Wang X. Lan T. (2010). Preparative isolation and purification of the active components from Viticis Fructus by high-speed counter-current chromatography. Sepu Chin. J. Chromatogr. 28, 1043–1047. 21381420

[B37] Gu Q. Zhang X. M. Jiang Z. Y. Chen J. J. Zhou J. (2007). Chemical constituents from fruits of *Vitex trifolia* . Chin. Tradit. Herb. Drugs 38, 656–659.

[B38] Gu Q. Zhang X.-M. Zhou J. Qiu S.-X. Chen J.-J. (2008). One new dihydrobenzofuran lignan from *Vitex trifolia* . J. Asian Nat. Prod. Res. 10, 499–502. 10.1080/10286020801967359 18470800

[B39] Hansel R. Leuckert C. Rimpler H. Schaaf K. (1965). Chemotaxomic investigation of the genus *Vitex* L. Phytochemistry 4, 19–27. 10.1016/s0031-9422(00)86142-7

[B40] Haripyaree A. Guneshwor K. Damayanti M. (2021). Antifungal and cytotoxic activities of five traditionally used indian medicinal plants. J. Microbiol. Biotechnol. Food Sci. 2021, 2272–2278.

[B41] Hashempur M. H. Mosavat S. H. Heydari M. Shams M. (2018). Medicinal plants’ use among patients with dyslipidemia: an Iranian cross-sectional survey. J. Complement. Integr. Med. 16, 20180101. 10.1515/jcim-2018-0101 30391934

[B42] Hernandez M. Heraso C. Villarreal M. Vargas-Arispuro I. Aranda E. (1999). Biological activities of crude plant extracts from *Vitex trifolia* L.(Verbenaceae). J. Ethnopharmacol. 67, 37–44. 10.1016/s0378-8741(99)00041-0 10616958

[B43] Hossain M. Paul N. Sohrab M. Rahman E. Rashid M. (2001). Antibacterial activity of *Vitex trifolia* . Fitoterapia 72, 695–697. 10.1016/s0367-326x(01)00304-5 11543973

[B44] Huang M.-Y. Zhong L.-J. Xie J.-M. Wang F. Zhang Y.-H. (2013). A new taraxastane-type triterpene from *Vitex trifolia* var. simplicifolia. Helv. Chim. Acta 96, 2040–2045. 10.1002/hlca.201200614

[B45] Ikawati Z. Hertiani T. Izzati F. (2019). Immunomodulatory activity of an Indonesian herbal formulation for respiratory disorder. Pharmacogn. Mag. 15, 130. 10.4103/pm.pm_314_18

[B46] Ikawati Z. Wahyuono S. Maeyama K. (2001). Screening of several Indonesian medicinal plants for their inhibitory effect on histamine release from RBL-2H3 cells. J. Ethnopharmacol. 75, 249–256. 10.1016/s0378-8741(01)00201-x 11297859

[B47] Jangwan J. S. Aquino R. P. Mencherini T. Picerno P. Singh R. (2013). Chemical constituents of ethanol extract of leaves and molluscicidal activity of crude extracts from *Vitex trifolia* Linn. Herba Pol. 59, 19–32. 10.2478/hepo-2013-0021

[B48] Kamal N. Mio Asni N. S. Rozlan I. N. A. Mohd Azmi M. A. H. Mazlan N. W. Mediani A. (2022). Traditional medicinal uses, phytochemistry, biological properties, and health applications of vitex sp. Plants 11, 1944. 10.3390/plants11151944 35893648 PMC9370779

[B49] Kannathasan K. Senthilkumar A. Chandrasekaran M. Venkatesalu V. (2007). Differential larvicidal efficacy of four species of Vitex against *Culex quinquefasciatus* larvae. Parasitol. Res. 101, 1721–1723. 10.1007/s00436-007-0714-5 17701216

[B50] Kannathasan K. Senthilkumar A. Venkatesalu V. (2011). Mosquito larvicidal activity of methyl-p-hydroxybenzoate isolated from the leaves of *Vitex trifolia* Linn. Acta Trop. 120, 115–118. 10.1016/j.actatropica.2011.07.001 21763671

[B51] Kannathasan K. Senthilkumar A. Venkatesalu V. Chandrasekaran M. (2008). Larvicidal activity of fatty acid methyl esters of Vitex species against *Culex quinquefasciatus* . Parasitol. Res. 103, 999–1001. 10.1007/s00436-008-1078-1 18553188

[B52] Karakoti H. Mahawer S. K. Tewari M. Kumar R. Prakash O. De Oliveira M. S. (2022). Phytochemical profile, *in vitro* bioactivity evaluation, *in silico* molecular docking and ADMET study of essential oils of three vitex species grown in tarai region of uttarakhand. Antioxidants 11, 1911. 10.3390/antiox11101911 36290633 PMC9598352

[B53] Kirtikar BASU Kirtikar (1980). Indian medicinal plant. 2nd Edn. Dehradun, Delhi, India: Bishen Singh Mahendra Pal Singh.

[B54] Kiuchi F. Matsuo K. Ito M. Qui T. K. Honda G. (2004). New norditerpenoids with trypanocidal activity from *Vitex trifolia* . Chem. Pharm. Bull. 52, 1492–1494. 10.1248/cpb.52.1492 15577254

[B55] Kulkarni L. (2011). Anti-inflammatory activity of *Vitex trifolia* Linn.(verbaneaceae) leaves extracts. Int. J. Pharm. Sci. Res. 2, 2037. 10.13040/IJPSR.0975-8232.2(8).2037-40

[B56] Kumar-Roine S. Matsui M. Reybier K. Darius H. T. Chinain M. Pauillac S. (2009). Ability of certain plant extracts traditionally used to treat ciguatera fish poisoning to inhibit nitric oxide production in RAW 264.7 macrophages. J. Ethnopharmacol. 123, 369–377. 10.1016/j.jep.2009.03.039 19501268

[B57] Lee H. G. Kim T. Y. Jeon J. H. Lee H. S. Hong Y. K. Jin M. H. (2016). Inhibition of melanogenesis by abietatriene from *Vitex trifolia* leaf oil. Nat. Prod. Sci. 22, 252–258. 10.20307/nps.2016.22.4.252

[B58] Lee C. Lee J. W. Jin Q. Lee H. J. Lee S. J. Lee D. (2013). Anti-inflammatory constituents from the fruits of *Vitex rotundifolia* . Bioorg Med. Chem. Lett. 23, 6010–6014. 10.1016/j.bmcl.2013.08.004 24035341

[B59] Li W.-X. Cui C.-B. Cai B. Wang H.-Y. Yao X.-S. (2005a). Flavonoids from *Vitex trifolia* L. inhibit cell cycle progression at G2/M phase and induce apoptosis in mammalian cancer cells. J. Asian Nat. Prod. Res. 7, 615–626. 10.1080/10286020310001625085 16087636

[B60] Li W. X. Cui C. B. Cai B. Wang H. Y. Yao X. S. (2005c). Flavonoids from *Vitex trifolia* L. inhibit cell cycle progression at G2/M phase and induce apoptosis in mammalian cancer cells. J. Asian Nat. Prod. Res. 7, 615–626. 10.1080/10286020310001625085 16087636

[B61] Li W.-X. Cui C.-B. Cai B. Yao X.-S. (2005b). Labdane-type diterpenes as new cell cycle inhibitors and apoptosis inducers from *Vitex trifolia* L. J. Asian Nat. Prod. Res. 7, 95–105. 10.1080/10286020310001617165 15621610

[B62] Li X. X. Wang L. Liu Y. L. Zhao Z. X. Wang X. L. Lei R. (2020a). Comprehensive identification of *Vitex trifolia* fruit and its five adulterants by comparison of micromorphological, microscopic characteristics, and chemical profiles. Microsc. Res. Tech. 83, 1530–1543. 10.1002/jemt.23547 32734676

[B63] Li X. X. Wang L. Liu Y. L. Zhao Z. X. Wang X. L. Lei R. (2020b). Comprehensive identification of *Vitex trifolia* fruit and its five adulterants by comparison of micromorphological, microscopic characteristics, and chemical profiles. MRT 83, 1530–1543. 10.1002/jemt.23547 32734676

[B64] Limousin P. Bessieres E. (2006). Oceania Planta Medica: flore de Kanaky, Tome I, Au bord de mer. Poindimié, Nouvelle-Caledonie: Bibliothèque de Nouméa, 102–104.

[B65] Liou C.-J. Cheng C.-Y. Yeh K.-W. Wu Y.-H. Huang W.-C. (2018). Protective effects of casticin from *Vitex trifolia* alleviate eosinophilic airway inflammation and oxidative stress in a murine asthma model. Front. Pharmacol. 9, 635. 10.3389/fphar.2018.00635 29962952 PMC6010522

[B66] Liu Q.-Y. Chen Y.-S. Wang F. Chen S.-W. Zhang Y.-H. (2014). Chemical of *Vitex trifolia* . China J. Chin. Mater Med. 39, 2024–2028. 25272835

[B67] Liu W. Feng Y. Yu S. Fan Z. Li X. Li J. (2021). The flavonoid biosynthesis network in plants. Int. J. Mol. Sci. 22, 12824. 10.3390/ijms222312824 34884627 PMC8657439

[B68] Luo P. Xia W. Morris-Natschke S. L. Lee K.-H. Zhao Y. Gu Q. (2017b). Vitepyrroloids A–D, 2-cyanopyrrole-containing labdane diterpenoid alkaloids from the leaves of *Vitex trifolia* . J. Nat. Prod. 80, 1679–1683. 10.1021/acs.jnatprod.6b01195 28453270 PMC5520631

[B69] Luo P. Xia W. Morris-Natschke S. L. Lee K.-H. Zhao Y. Gu Q. (2017a). Vitepyrroloids A–D, 2-cyanopyrrole-containing labdane diterpenoid alkaloids from the leaves of *Vitex trifolia* . J. Nat. Prod. 80, 1679–1683. 10.1021/acs.jnatprod.6b01195 28453270 PMC5520631

[B70] Luo P. Yu Q. Liu S.-N. Xia W.-J. Fang Y.-Y. An L.-K. (2017c). Diterpenoids with diverse scaffolds from *Vitex trifolia* as potential topoisomerase I inhibitor. Fitoterapia 120, 108–116. 10.1016/j.fitote.2017.06.006 28602939

[B71] Maffei M. E. Gertsch J. Appendino G. (2011). Plant volatiles: production, function and pharmacology. Nat. Prod. Rep. 28, 1359–1380. 10.1039/c1np00021g 21670801

[B72] Manaf S. R. Daud H. M. (2016). Screening of phytochemical properties and antimicrobial activity of Malaysian medicinal plants against aquatic bacteria. Malays. J. Microbiol., 10.21161/mjm.83816

[B73] Manjunatha B. K. Vidya S. M. (2008). Hepatoprotective activity of *Vitex trifolia* against carbon tetrachloride-induced hepatic damage. Indian J. Pharm. Sci. 70, 241–245. 10.4103/0250-474X.41466 20046723 PMC2792475

[B74] Manjunatha B. K. Vidya S. M. Krishna V. Mankani K. L. Singh S. D. J. Manohara Y. N. (2007). Comparative evaluation of wound healing potency of *Vitex trifolia* L. and *Vitex altissima* L. Phytother. Res. 21, 457–461. 10.1002/ptr.2094 17262889

[B75] Mathankumar M. Tamizhselvi R. Manickam V. Purohit G. (2015). Assessment of anticarcinogenic potential of *Vitex trifolia* and *Triticum aestivum* linn by *in vitro* rat liver microsomal degranulation. Toxicol. Int. 22, 114–118. 10.4103/0971-6580.172269 26862271 PMC4721158

[B76] Matsui M. Adib-Conquy M. Coste A. Kumar-Roine S. Pipy B. Laurent D. (2012). Aqueous extract of *Vitex trifolia* L. (Labiatae) inhibits LPS-dependent regulation of inflammatory mediators in RAW 264.7 macrophages through inhibition of Nuclear Factor kappa B translocation and expression. J. Ethnopharmacol. 143, 24–32. 10.1016/j.jep.2012.05.043 22732725

[B77] Matsui M. Kumar-Roine S. Darius H. T. Chinain M. Laurent D. Pauillac S. (2009). Characterisation of the anti-inflammatory potential of *Vitex trifolia* L. (Labiatae), a multipurpose plant of the Pacific traditional medicine. J. Ethnopharmacol. 126, 427–433. 10.1016/j.jep.2009.09.020 19778597

[B78] Meng Z.-L. Qi Y.-Y. Liu R.-M. Sun A.-L. Wang D.-Q. (2006). 5, 12-Dihydroxy-2, 6, 7, 13-tetramethoxyflavone. Acta Crystallogr. Sect. E Struct. Rep. 62, o3831–o3832. 10.1107/s1600536806031175

[B79] Mohamed M. Abdou A. Hamed M. Saad A. (2013). Characterization of bioactive phytochemical from the leaves of *Vitex trifolia* . Int. J. Pharm. Appl. 3, 419–428.

[B80] Mohanbabu A. V. Kishore M. K. Chandrashekar B. R. Pradeepa H. D. Christopher R. Nandit P. B. (2015). Evaluation of potential antiamnesic activities of aqueous extract of *Vitex trifolia* leaves against scopolamine induced amnesia and in normal rats. J. Basic Clin. Physiol. Pharmacol. 26, 201–209. 10.1515/jbcpp-2014-0002 25153588

[B81] Mosavat S. H. Ghahramani L. Haghighi E. R. Chaijan M. R. Hashempur M. H. Heydari M. (2015). Anorectal diseases in avicenna’s “canon of medicine”. Acta medico-historica Adriat. AMHA 13, 103–114. 26959635

[B82] Mottaghipisheh J. Taghrir H. Boveiri Dehsheikh A. Zomorodian K. Irajie C. Mahmoodi Sourestani M. (2021). Linarin, a glycosylated flavonoid, with potential therapeutic attributes: a comprehensive review. Pharmaceuticals 14, 1104. 10.3390/ph14111104 34832886 PMC8621830

[B83] Musa N. Banerjee S. Maspalma G. Usman L. Hussaini B. (2022). Assessment of the phytochemical, antioxidant and larvicidal activity of essential oil extracted from Simpleleaf Chastetree [*Vitex trifolia*] leaves obtained from Ganye Local Government, Adamawa State-Nigeria. Mat. Today. 49, 3435–3438. 10.1016/j.matpr.2021.03.375

[B84] Nabavi S. M. Šamec D. Tomczyk M. Milella L. Russo D. Habtemariam S. (2020). Flavonoid biosynthetic pathways in plants: versatile targets for metabolic engineering. Biotechnol. Adv. 38, 107316. 10.1016/j.biotechadv.2018.11.005 30458225

[B85] Nair A. R. Ramesh P. Subramanian S. S. (1975). Two unusual flavones (artemetin and 7-desmethyl artemetin) from the leaves of *Vitex trifolia* . Curr. Sci., 214–216.

[B86] Natheer S. E. Sekar C. Amutharaj P. Rahman M. S. A. Khan K. F. (2012). Evaluation of antibacterial activity of *Morinda citrifolia*, *Vitex trifolia* and Chromolaena odorata. AJPP 6, 783–788. 10.5897/ajpp11.435

[B87] Nishina A. Itagaki M. Sato D. Kimura H. Hirai Y. Phay N. (2017). The rosiglitazone-like effects of vitexilactone, a constituent from *Vitex trifolia* L. in 3T3-L1 preadipocytes. Molecules 22, 2030. 10.3390/molecules22112030 29165364 PMC6150318

[B88] Nyamoita M. G. Ester I. Zakaria M. H. Wilber L. Bwire O. J. Ahmed H. (2013). Comparison of the effects of extracts from three Vitex plant species on *Anopheles gambiae* s.s. (Diptera: Culicidae) larvae. Acta Trop. 127, 199–203. 10.1016/j.actatropica.2013.05.003 23688936

[B89] Ono M. Ito Y. Nohara T. (2001). Four new halimane-type diterpenes, vitetrifolins D-G, from the fruit of *Vitex trifolia* . Chem. Pharm. Bull. (Tokyo) 49, 1220–1222. 10.1248/cpb.49.1220 11558619

[B90] Ono M. Sawamura H. Ito Y. Mizuki K. Nohara T. (2000). Diterpenoids from the fruits of *Vitex trifolia* . Phytochemistry 55, 873–877. 10.1016/s0031-9422(00)00214-4 11140517

[B91] Pan J. Xu Z. Fan J. (1989). GC-MS analysis of essential oils from four *Vitex* species. Zhongguo Zhongyao Zazhi 14, 357–359. 2511861

[B92] Peng X. Sun F. Li G. Wang C. Zhang Y. Wu C. (2021). New xanthones with antiagricultural fungal pathogen activities from the endophytic fungus Diaporthe goulteri L17. J. Agric. Food Chem. 69, 11216–11224. 10.1021/acs.jafc.1c03513 34541846

[B93] Prasad A. Devi A. T. Prasad M. N. N. Zameer F. Shruthi G. Shivamallu C. (2019). Phyto anti-biofilm elicitors as potential inhibitors of *Helicobacter pylori* . 3 Biotech. 9, 53. 10.1007/s13205-019-1582-2 PMC6349266 30729077

[B94] Quan-Yu Liu Y.-S. C. Wang F. E. I. Chen S. H. I.-W. U. Zhang YONG.-HONG (2014). Chemical of *Vitex trifolia* . Zhongguo Zhong Yao Za Zhi 39, 2024–2228. 25272835

[B95] Rani A. Sharma A. (2013). The genus Vitex: a review. Pharmacogn. Rev. 7, 188–198. 10.4103/0973-7847.120522 24347927 PMC3841997

[B96] Roncero A. M. Tobal I. E. Moro R. F. Diez D. Marcos I. S. (2018). Halimane diterpenoids: sources, structures, nomenclature and biological activities. Nat. Prod. Rep. 35, 955–991. 10.1039/c8np00016f 29701206

[B97] Saklani S. Mishra A. P. Chandra H. Atanassova M. S. Stankovic M. Sati B. (2017). Comparative evaluation of polyphenol contents and antioxidant activities between ethanol extracts of *Vitex negundo* and *Vitex trifolia* L. Leaves by different methods. Plants 6, 45. 10.3390/plants6040045 28953235 PMC5750621

[B98] Salah E.-K. Mohamed M. Mohamed S. (2012). Phenolic and biological activities of *Vitex trifolia* aerials parts. Life Sci. J. 9, 670–677.

[B99] Sena Filho J. G. Duringer J. Maia G. L. Tavares J. F. Xavier H. S. Da Silva M. S. (2008). Ecdysteroids from Vitex species: distribution and compilation of their 13C-NMR spectral data. Chem. Biodivers. 5, 707–713. 10.1002/cbdv.200890067 18493957

[B100] Shah A. Rahim S. (2017). Ethnomedicinal uses of plants for the treatment of malaria in Soon Valley, Khushab, Pakistan. J. Ethnopharmacol. 200, 84–106. 10.1016/j.jep.2017.02.005 28192202

[B101] Shah S. Dhanani T. Kumar S. (2013). Validated HPLC method for identification and quantification of p-hydroxy benzoic acid and agnuside in *Vitex negundo* and *Vitex trifolia* . J. Pharm. Anal. 3, 500–508. 10.1016/j.jpha.2013.09.008 29403861 PMC5761016

[B102] Suchitra M. Cheriyan B. V. (2018). *Vitex trifolia*: an ethnobotanical and pharmacological review. Asian J. Pharm. Clin. Res. 11, 12–14. 10.22159/ajpcr.2018.v11s4.31689

[B103] Sukarsih Y. Arfiansyah R. Roska T. P. Murdifin M. Kasim S. Nainu F. (2021). Protective effect of ethanol extract of legundi (*Vitex trifolia* L.) leaves against *Staphylococcus aureus* in Drosophila infection model. Biointerface Res. Appl. Chem. 11, 13989–13996. 10.33263/BRIAC116.1398913996

[B104] Suksamrarn A. Werawattanametin K. Brophy J. J. (1991). Variation of essential oil constituents in *Vitex trifolia* species. Flavour Fragr. J. 6, 97–99. 10.1002/ffj.2730060115

[B105] Talreja S. Tiwari S. (2020). Medicinal and pharmacological importance of *Vitex trifolia*: a review. Res. J. Pharm. Biol. Chem. Sci. 11, 8–13. 10.33887/rjpbcs/2020.11.5.2

[B106] Tandon S. Mittal A. K. Pant A. K. (2008). Insect growth regulatory activity of *Vitex trifolia* and *Vitex agnus-castus* essential oils against Spilosoma obliqua. Fitoterapia 79, 283–286. 10.1016/j.fitote.2007.11.032 18353565

[B107] Tawatsin A. Asavadachanukorn P. Thavara U. Wongsinkongman P. Bansidhi J. Boonruad T. (2006). Repellency of essential oils extracted from plants in Thailand against four mosquito vectors (Diptera: Culicidae) and oviposition deterrent effects against *Aedes aegypti* (Diptera: Culicidae). Southeast Asian J. Trop. Med. Public Health. 37, 915–931. 17333734

[B108] Thenmozhi S. Lakshmia A. Priya L. Dwivedi S. (2015). Comparative pharmacognostical and phytochemical evaluation between the leaves of *Vitex trifolia* linn. And *Vitex leucoxylon* linn. Int. J. Mol. Sci. 6, 2120–2126.

[B109] Thoa N. T. K. Ban N. K. Trang D. T. Linh T. M. Giang V. H. Nhiem N. X. (2018). Ecdysteroids from leaves of *Vitex trifolia* . Vietnam J. Chem. 56, 162–166. 10.1002/vjch.201800006

[B110] Thomas R. Ramachandran A. Paul J. Mohan M. (2019). Essential oils studies of the genus *Vitex* L.(Verbenaceae). Int. J. Adv. Res. 7, 568–574. 10.21474/ijar01/9074

[B111] Tiwari N. Luqman S. Masood N. Gupta M. M. (2012). Validated high performance thin layer chromatographic method for simultaneous quantification of major iridoids in *Vitex trifolia* and their antioxidant studies. J. Pharm. Biomed. Anal. 61, 207–214. 10.1016/j.jpba.2011.12.007 22226914

[B112] Tiwari N. Thakur J. Saikia D. Gupta M. M. (2013). Antitubercular diterpenoids from *Vitex trifolia* . Phytomedicine 20, 605–610. 10.1016/j.phymed.2013.01.003 23462211

[B113] Tiwari N. Yadav D. Singh S. Gupta M. (2011). A marker-based stability indicating high performance thin layer chromatographic method for *Vitex trifolia* . J. Liq. Chromatogr. Relat. Technol. 34, 1925–1937. 10.1080/10826076.2011.582213

[B114] Ukiya M. Sato D. Kimura H. Koketsu M. Phay N. Nishina A. (2019a). (-)-O-Methylcubebin from *Vitex trifolia* enhanced adipogenesis in 3T3-L1 cells via the inhibition of ERK1/2 and p38MAPK phosphorylation. Molecules 25, 73. 10.3390/molecules25010073 31878261 PMC6994966

[B115] Ukiya M. Sato D. Kimura H. Koketsu M. Phay N. Nishina A. (2019b). (-)-O-Methylcubebin from *Vitex trifolia* enhanced adipogenesis in 3T3-L1 cells via the inhibition of ERK1/2 and p38MAPK phosphorylation. Molecules 25, 73. 10.3390/molecules25010073 31878261 PMC6994966

[B116] Villasenor M. I. (2007). Bioactivities of iridoids. Antiinflamm. Antiallergy Agents Med. Chem. 6, 307–314. 10.2174/187152307783220040

[B117] Vimalanathan S. Ignacimuthu S. Hudson J. B. (2009). Medicinal plants of Tamil Nadu (Southern India) are a rich source of antiviral activities. Pharm. Biol. 47, 422–429. 10.1080/13880200902800196

[B118] Wang H. Y. Cai B. Cui C. B. Zhang D. Y. Yang B. F. (2005). Vitexicarpin, a flavonoid from *Vitex trifolia* L., induces apoptosis in K562 cells via mitochondria-controlled apoptotic pathway. Yao Xue Xue Bao 40, 27–31. 15881322

[B119] Wee H.-N. Neo S.-Y. Singh D. Yew H.-C. Qiu Z.-Y. Tsai X.-R. C. (2020). Effects of *Vitex trifolia* L. leaf extracts and phytoconstituents on cytokine production in human U937 macrophages. BMC Complement. Med. Ther. 20, 91. 10.1186/s12906-020-02884-w 32188443 PMC7081688

[B120] Winarno E. K. Susanto Winarno H. (2020). Antiproliferative activity against cancer cell lines of gamma irradiated “Legundi” (*Vitex Trifolia* L.) leaves and its chromatogram profiles. AIP Conf. Proc. 2296, 020068. 10.1063/5.0030628

[B121] Woradulayapinij W. Soonthornchareonnon N. Wiwat C. (2005). *In vitro* HIV type 1 reverse transcriptase inhibitory activities of Thai medicinal plants and Canna indica L. rhizomes. J. Ethnopharmacol. 101, 84–89. 10.1016/j.jep.2005.03.030 15951145

[B122] Wu J. Zhou T. Zhang S.-W. Zhang X.-H. Xuan L.-J. (2009a). Cytotoxic terpenoids from the fruits of *Vitex trifolia* L. Planta medica. 75, 367–370. 10.1055/s-0028-1112211 19156599

[B123] Wu J. Zhou T. Zhang S. W. Zhang X. H. Xuan L. J. (2009b). Cytotoxic terpenoids from the fruits of *Vitex trifolia* L. Planta Med. 75, 367–370. 10.1055/s-0028-1112211 19156599

[B124] Yahaya M. F. Yelwa J. M. Abdullahi S. Umar J. B. Abubakar A. M. Babakura M. (2019). Chemical compositions, FTIR and larvicidal activity of essential oils extracted from aromatic plants. Eur. Sci. J. ESJ 15, 110. 10.19044/esj.2019.v15n24p110

[B125] Yan C.-X. Wei Y.-W. Li H. Xu K. Zhai R.-X. Meng D.-C. (2023). Vitex rotundifolia L. f. and *Vitex trifolia* L.: a review on their traditional medicine, phytochemistry, pharmacology. J. Ethnopharmacol. 308, 116273. 10.1016/j.jep.2023.116273 36822343

[B126] Yao J.-L. Fang S.-M. Liu R. Oppong M. B. Liu E.-W. Fan G.-W. (2016). A review on the terpenes from genus vitex. Molecules 21, 1179. 10.3390/molecules21091179 27608002 PMC6273030

[B127] Yong-Sheng Chen J.-M. X. Hong Y. A. O. Xiao-Yan L. I. N. Zhang YONG.-HONG Zhang Y. H. (2010). Studies on the triterpenoids of *Vitex trifolia* . Zhong Yao Cai 33, 908–910. 21049612

[B128] Zaki F. L. M. Salleh W. Noor N. N. M. Shaharudin S. M. Ab Ghani N. (2022). Characterisation of the essential oil components and their multivariate statistical analysis of the genus *Vitex* and *Plectranthus* (Lamiaceae). Riv. Ital. Sostanze Grasse 99, 263–268.

[B129] Zeng X. Fang Z. Wu Y. Zhnhg H. (1996). Chemical constituents of the fruits of *Vitex trifolia* L. Zhongguo Zhongyao Zazhi 21, 167–168. 9206258

[B130] Zheng C.-J. Zhu J.-Y. Yu W. Ma X.-Q. Rahman K. Qin L.-P. (2013a). Labdane-type diterpenoids from the fruits of *Vitex trifolia* . J. Nat. Prod. 76, 287–291. 10.1021/np300679x 23327905

[B131] Zheng H. Zhang W. Xu F. Zhang H. Chen X. Rui Y. (2013b). Study on volatile components of butterfly nectar plants and host plants. Asian. J. Chem. 25, 7861–7863. 10.14233/ajchem.2013.14659

[B132] Zhou L. Wang Y. Peng Z. J. S. S. S. 2009. Advances in study on formation mechanism and genetic engineering of yellow flowers. Scientia Silvae Sinicae. 12 (2), 111–119. 10.11707/j.1001-7488.20090219

