# Supplementary material for: A comprehensive review of ethnomedicinal approaches, phytochemical analysis, and pharmacological potential of Vitex trifolia L
Source: Front Pharmacol. 2024 Mar 21;15:1322083. doi: 10.3389/fphar.2024.1322083 (PMC10991721; doi:10.3389/fphar.2024.1322083)
Supplement: Supplementary file 1 [file Table1.docx]

**Table S1.** Secondary metabolites isolated from *V. trifolia*

| **No.** | **Metabolites** | **Classification** | **Plant part** | **Plant product: extract/EO** | **Chromatographic techniques** | **References** |
| --- | --- | --- | --- | --- | --- | --- |
| **Terpenoids** | | | | | | |
| **1** | **agnuside** | *iridoid* | *leaves* | MeOH extract  *n*-BuOH fraction | Silica gel column | (Tiwari et al., 2013) |
|  |  |  | *leaves and bark* | MeOH extract  HX, CHL, EtOAc , Aq fraction | RP-18 column | (Dhanani et al., 2015) |
|  |  |  | *aerial parts* | MeOH extract  *n-*BuOH fraction | Silica gel 60-column | (Tiwari et al., 2012) |
|  |  |  | *bark and leaves* | MeOH extract | HPLC method | (Shah et al., 2013) |
|  |  |  | *fruit* | MeOH extract | Ultra-performance liquid chromatography coupled with Orbitrap mass spectrometry (UPLC-Orbitrap-MS) | (Li et al., 2020b) |
| **2** | **10-*O*-vanilloylaucubin** |  | *fruit* | 80% EtOH extract | Silica gel column and HPLC | (Bao et al., 2018) |
| **3** | **(1S, 5S,6R,9R)-10-*O*-*p*-hydroxybenzoyl-5,6*β*-dihydroxy iridoid 1-*O*-*β*-*D*-glucopyranoside** |  | *fruits* | 80% EtOH extract | Silica gel, Sephadex LH-20, ODS open column chromatography | (Bao et al., 2018) |
| **4** | **mussaenosidic acid** |  | *leaves* | MeOH extract  *n*-BuOH fraction | Silica gel column, preparative HPLC | (Bao et al., 2018) |
| **5** | **negundoside** |  | *leaves* | MeOH extract  *n*-BuOH fraction | Silica gel column, preparative HPLC | (Bao et al., 2018) |
|  |  |  | *leaves and bark* | MeOH extract  HX, CHL, EtOAc , Aq fraction | RP-18 | (Bao et al., 2018) |
|  |  |  | *aerial parts* | MeOH extract  *n-BuOH* fraction | Silica gel column, preparative HPLC | (Bao et al., 2018) |
| **6** | **agnusoside** |  | *fruits* | 80% EtOH extract | Silica gel column and HPLC | (Bao et al., 2018) |
| **7** | **nishindaside** |  | *fruits* | 80% EtOH extract | Silica gel column chromatography, and HPLC | (Bao et al., 2018) |
| **8** | **3-normal-butyl-nishindaside** |  | *fruits* | 80% EtOH extract  *n*-BuOH fraction | Silica gel and ODS open column chromatography, and HPLC | (Bao et al., 2018) |
| **9** | **3-normal-butyl-isonishindaside** |  | *fruits* | 80% EtOH extract  *n*-BuOH fraction | Silica gel and ODS open column chromatography, and HPLC | (Bao et al., 2018) |
| **10** | **6-*p*-hydroxy benzoyl mussaenosidic acid** |  | *aerial parts* | MeOH extract  *n*-BuOH fraction | Silica gel, preparative HPLC | (Tiwari et al., 2012) |
|  |  |  | *leaves* | MeOH extract  *n*-BuOH fraction | Silica gel column, preparative HPLC | (Tiwari et al., 2013) |
| **11** | **viteoid II** |  | *fruit* | MeOH extract | Utra-performance liquid chromatography coupled with Orbitrap mass spec-  trometry (UPLC-Orbitrap-MS) | (Li et al., 2020b) |
| **12** | **eucommiol** |  | *fruits* | *n*-BuOH extract | Silica gel column chromatography | (Gu et al., 2008) |
| **13** | **pedicularis lactone** | *iridoid lactone* | *fruit* | MeOH extract | ultra-performance liquid chromatography coupled with Orbitrap mass spec-  trometry (UPLC-Orbitrap-MS) | (Li et al., 2020b) |
| **14** | **viteoid I** |  | *fruit* | MeOH extract | Ultra-performance liquid chromatography coupled with Orbitrap mass spec-  trometry (UPLC-Orbitrap-MS) | (Li et al., 2020b) |
| **15** | **vitexoid** | *acyclic monoterpenoid* | *fruits* | 95% EtOH extract  EtOAc fraction | Silica gel column chromatography (CC) and Sephadex LH-20, MCI gel | (Djimabi et al., 2021) |
|  |  |  |  | 30% Ace  CHL fraction | Silica gel, MCI CHP-20P, Cosmosil 75 C18-OPN, Toyopearl HW-40F, Sephadex LH-20, Al2O3 | (Wu et al., 2009a) |
| **16** | **spathulenol** | *Sesquiterpenoids* | *fruits* | 95% EtOH extract | Silica gel, Sephadex LH-20, and Rp-8 and Rp-18 gel column chromatography | (Gu et al., 2007) |
| **17** | ***ent*-4*α*,10*β*-dihydroxyaromadendrane** |  | *fruits* | 95% EtOH extract | Silica gel, Sephadex LH-20, and Rp-8 and Rp-18 gel column chromatography | (Gu et al., 2007) |
| **Diterpenoids** | | | | | | |
| **18** | **vitextrifolin A** | *Labdane* | *fruits* | 80% EtOH extract  DCM fraction | Silica gel, Sephadex LH20 and MCI gel column chromatography, preparative TLC | (Zheng et al., 2013a) |
| **19** | **vitextrifolin B** |  | *fruits* | 80% EtOH extract  DCM fraction | Silica gel, Sephadex LH20 and MCI gel column chromatography, preparative TLC | (Zheng et al., 2013a) |
| **20** | **vitetrolin C** |  | *fruits* | 95% EtOH Extract  EtOAc fraction | MCI gel CC (MeOH - H_2_O, 3:7 to 1:0), silica gel CC eluted with petroleum ether - EtOAc (20:1 to 1:1), ODS C18 column (MeOH - H_2_O, 3:7–1:0), preparative TLC (CH_2_Cl_2_ - acetone 10:1) | (Djimabi et al., 2022) |
| **21** | **vitextrifolin C** |  | *fruits* | 80% EtOH extract  DCM fraction | Silica gel and MCI gel column chromatography, semipreparative HPLC | (Zheng et al., 2013a) |
|  |  |  | *leaves* | 95% EtOH extract  EtOAc fraction | Silica gel, RP-C18 and MCI gel column chromatography, semi-preparative HPLC | (Luo et al., 2017c) |
| **22** | **vitextrifolin D** |  | *fruits* | 80% EtOH extract  DCM fraction | Silica gel, Sephadex LH20 and MCI gel column chromatography | (Zheng et al., 2013a) |
| **23** | **vitextrifolin E** |  | *fruits* | 80% EtOH extract  DCM fraction | Silica gel and MCI gel column chromatography, semi preparative HPLC | (Zheng et al., 2013a) |
| **24** | **vitextrifolin F** |  | *fruits* | 80% EtOH extract  DCM fraction | Silica gel and MCI gel column chromatography | (Zheng et al., 2013a) |
| **25** | **vitextrifolin G** |  | *fruits* | 80% EtOH extract  DCM fraction | Silica gel and MCI gel column chromatography | (Zheng et al., 2013a) |
| **26** | **rotundifuran** |  | *fruits* | 80% EtOH extract  DCM fraction | Silica gel and Sephadex LH20 column chromatography, semi preparative HPLC | (Zheng et al., 2013a) |
|  |  |  | *fruits* | Ace extract  Hx fraction | Silica gel column chromatography (CC) and HPLC | (Djimabi et al., 2021) |
|  |  |  | *fruits* | 95% EtOH extract  EtOAc fraction | Silica gel column chromatography | (Djimabi et al., 2021) |
|  |  |  | *fruits* | PE extracts | Vacuum liquid chromatography over silica gel H chromatography | (Li et al., 2005b) |
| **27** | **vitetrifolin B** |  | *fruits* | *Ace extract*  Hx fraction | Silica gel column chromatography and preparative TLC | (Djimabi et al., 2021) |
|  |  |  | *fruits* | 95% EtOH extract  EtOAc fraction | Silica gel column chromatography (CC) and Sephadex LH-20 | (Djimabi et al., 2021) |
| **28** | **dihydrosolidagenone** |  | *fruits* | Ace extract  Hx fraction | Silica gel column chromatography (CC) and HPLC | (Ono et al., 2000) |
|  |  |  | *fruits* | 95% EtOH extract  EtOAc fraction | Silica gel column chromatography (CC) and Sephadex LH-20 | (Djimabi et al., 2021) |
|  |  |  |  | 30% Ace  CHL fraction | Silica gel, MCI CHP-20P, Cosmosil 75 C18-OPN, Toyopearl HW-40F, Sephadex LH-20, Al2O3 column chromatography | (Wu et al., 2009a) |
| **29** | **15, 16-epoxy-9-hydroxylabda-13(16),14-diene** |  | *fruits* | 95% EtOH extract  EtOAc fraction | Silica gel column chromatography | (Djimabi et al., 2021) |
| **30** | **vitexilactone** |  | *fruits* | 80% EtOH extract  DCM fraction | Silica gel column | (Zheng et al., 2013a) |
|  |  |  | fruits | 95 and  60% EtOH extracts  EtOAc fraction | Silica gel column chromatography (CC) and, MCI gel CC, Preparative HPLC | (Fang et al., 2019) |
|  |  |  | fruits | 95% EtOH extract  CHCl3 fraction | Silica gel and ODS column chromatography | (Fang et al., 2019) |
|  |  |  | leaves | EtOAc extract | Silica gel column chromatography and ODS-HPLC | (Nishina et al., 2017) |
|  |  |  | leaves | 95% EtOH extract  EtOAc fraction | Silica gel and RP-C18 column | (Luo et al., 2017c) |
|  |  |  | fruits | CHL extracts | vacuum liquid chromatography over silica gel H, ODS column chromatography | (Li et al., 2005b) |
|  |  |  | *fruits* | Ace extract | silica gel column and RP-18 column chromatography | (Kiuchi et al., 2004) |
|  |  |  | *fruits* | 30% Ace  CHL fraction | silica gel, MCI CHP-20P, Cosmosil 75 C18-OPN, Toyopearl HW-40F, Sephadex LH-20, Al2O3 column chromatography | (Wu et al., 2009a) |
|  |  |  | *fruits* | 95% EtOH extract  EtOAc fraction | silica gel column chromatography (CC) and MCI gel | (Djimabi et al., 2021) |
|  |  |  | *fruits* | 95% EtOH extract | silica gel, Sephadex LH-20, and Rp-8 and Rp-18 gel column chromatography | (Gu et al., 2007) |
| **31** | **vitexilactone B** |  | *fruits* | 80% EtOH extract  DCM fraction | silica gel and MCI gel column chromatography | (Zheng et al., 2013a) |
|  |  |  | leaves | 95% EtOH extract  EtOAc fraction | semi-preparative HPLC, silica gel CC and Sephadex LH-20 column chromatography | (Luo et al., 2017c) |
| **32** | **deacetylvitexilactone** |  | *fruits* | 80% EtOH extract  DCM fraction | silica gel, Sephadex LH20 and MCI gel column chromatography | (Zheng et al., 2013a) |
|  |  |  | *fruit* | *MeOH extract* | ultra-performance liquid chromatography coupled with Orbitrap mass spec-  trometry (UPLC-Orbitrap-MS) | (Li et al., 2020b) |
|  |  |  | *leaves* | 95% EtOH extract  EtOAc fraction | silica gel and RP-C18 column, semi-preparative HPLC | (Luo et al., 2017c) |
| **33** | **viteagnusin I** |  | *fruits* | 80% EtOH extract  DCM fraction | silica gel, Sephadex LH20 and MCI gel column chromatography | (Zheng et al., 2013a) |
|  |  |  | *leaves* | 95% EtOH extract  EtOAc fraction | semi-preparative HPLC, silica gel CC and MCI gel column chromatography | (Luo et al., 2017c) |
| **34** | **viteosin A** |  | *leaves* | HX extract  Ace fraction | preparative TLC | (Alam et al., 2002) |
| **35** | **viterotulin B** |  | *leaves* | 95% EtOH extract  EtOAc fraction | silica gel CC and Sephadex LH-20 column chromatography , semi-preparative HPLC | (Luo et al., 2017c) |
|  |  |  | *fruit* | MeOH extract | ultra-performance liquid chromatography coupled with Orbitrap mass spec-  trometry (UPLC-Orbitrap-MS) | (Li et al., 2020b) |
| **36** | **9-hydroxy-13(14)-labden-15,16-olide** |  | *leaves* | MeOH extract  Hx fraction | silica gel column, preparative HPLC | (Tiwari et al., 2013) |
| **37** | **viterotulin D** |  | *fruits* | 95% EtOH extract  EtOAc fraction | silica gel column chromatography (CC) and ODS-C18, MCI  gel | (Djimabi et al., 2021) |
| **38** | **viterotulin C** |  | *fruits* | 95% EtOH extract  EtOAc fraction | silica gel column chromatography (CC) and Sephadex LH-20, MCI  gel | (Djimabi et al., 2021) |
| **39** | **6-acetoxy-9-hydroxy-13(14)-labdan-16,15-olide** |  | *fruits* | 30% Ace  CHL fraction | silica gel, MCI CHP-20P, Cosmosil 75 C18-OPN, Toyopearl HW-40F, Sephadex LH-20, Al2O3 | (Wu et al., 2009a) |
|  |  |  | *fruits* | Ace extract | silica gel column and RP-18 column chromatography | (Kiuchi et al., 2004) |
|  |  |  | *fruits* | CHL and PE extracts | vacuum liquid chromatography over silica gel H, ODS column chromatography | (Li et al., 2005b) |
| **40** | **viterotulin A** |  | *fruits* | 95% EtOH Extract  EtOAc fraction | silica gel column chromatography (CC) eluted with a gradient solvent system of petroleum ether - acetone (40:1 to 1:1, V/V), MCI gel CC (MeOH - H_2_O, 3:7 to 1:0), silica gel CC eluted with petroleum ether - EtOAc (20:1 to 1:1), Sephadex LH-20 column eluted with methanol | (Djimabi et al., 2022) |
| **41** | **previtexilactone** |  | *fruits* | 80% EtOH extract  DCM fraction | silica gel and MCI gel column chromatography | (Zheng et al., 2013a) |
|  |  |  | *fruits* | *Ace extract* | *silica gel column and RP-18 column chromatography* | (Kiuchi et al., 2004) |
|  |  |  | *Fruits* | 30% Ace  CHL fraction | silica gel, MCI CHP-20P, Cosmosil 75 C18-OPN, Toyopearl HW-40F, Sephadex LH-20, Al2O3 column chromatography | (Wu et al., 2009a) |
|  |  |  | *fruits* | 95% EtOH extract  EtOAc fraction | silica gel column chromatography (CC) and MCI  gel | (Djimabi et al., 2021) |
|  |  |  | *leaves* | 95% EtOH extract  EtOAc fraction | silica gel CC and RP-C18 column | (Luo et al., 2017c) |
| **42** | **vitextrifloxide C** |  | *leaves* | 95% EtOH extract  EtOAc fraction | silica gel CC and RP-C18 column | (Luo et al., 2017c) |
| **43** | **negundol** |  | *fruits* | 80% EtOH extract  DCM fraction | silica gel and MCI gel column chromatography | (Zheng et al., 2013a) |
| **44** | **vitetrifolin C** |  | *fruit* | Ace extract  Hx fraction | silica gel column chromatography and ODS-HPLC | (Ono et al., 2000) |
| **45** | **6*α*,7*α*-diacetoxy-13-hydroxy-8(9),14-labdadien** |  | *leaves* | MeOH extract  Hx fraction | silica gel column, preparative HPLC | (Tiwari et al., 2013) |
| **46** | **manool** |  | *fruits* | 95% EtOH Extract  EtOAc fraction | silica gel column chromatography (CC) petroleum ether - acetone (40:1 to 1:1, V/V), MCI gel CC (MeOH - H_2_O, 3:7 to 1:0, V/V), silica gel CC (petroleum ether - EtOAc, 20:1 to 1:1), ODS C18, methanol aqueous solution (3:10–1:0), silica gel CC (CH_2_Cl_2_ - acetone, 100:1 to 5:1) | (Djimabi et al., 2022) |
| **47** | **vitetrolin A** |  | *fruits* | 95% EtOH Extract  EtOAc fraction | MCI gel CC (MeOH - H_2_O, 3:7 to 1:0), silica gel CC (petroleum ether - EtOAc, 10:1 to 1:1), Sephadex LH-20 (CHCl3 - MeOH, 2:3), preparative TLC (CH_2_Cl_2_ - acetone, 30:2) | (Djimabi et al., 2022) |
| **48** | **vitetrolin B** |  | *fruits* | 95% EtOH Extract  EtOAc fraction | MCI gel CC (MeOH - H_2_O, 3:7 to 1:0), silica gel CC eluted with petroleum ether - EtOAc (20:1 to 1:1), ODS C18 column (MeOH - H_2_O, 3:7–1:0), preparative TLC (CH_2_Cl_2_ - acetone 10:1) | (Djimabi et al., 2022) |
| **49** | **vitextrifloxide A** |  | *leaves* | 95% EtOH extract  EtOAc fraction | silica gel CC and RP-C18 column | (Luo et al., 2017c) |
| **50** | **vitextrifloxide B** |  | *leaves* | 95% EtOH extract  EtOAc fraction | semi-preparative HPLC, silica gel CC and RP-C18 column | (Luo et al., 2017c) |
| **51** | **vitextrifloxide D** |  | *leaves* | 95% EtOH extract  EtOAc fraction | silica gel CC and RP-C18 column | (Luo et al., 2017c) |
| **52** | **vitextrifloxide E** |  | *leaves* | 95% EtOH extract  EtOAc fraction | silica gel CC , RP-C18 column | (Luo et al., 2017c) |
| **53** | **(*rel* 5S,6R,8R,9R,10S,13S,15R) 6-acetoxy-9,13;15,16-diepoxy-15-methoxylabdane** |  | *fruits* | 30% Ace  CHL fraction | silica gel, MCI CHP-20P, Cosmosil 75 C18-OPN, Toyopearl HW-40F, Sephadex LH-20, Al2O3 | (Wu et al., 2009a) |
| **54** | **vitetrolin D** |  | *fruits* | 95% EtOH Extract  EtOAc fraction | MCI gel CC (MeOH - H_2_O, 3:7 to 1:0), silica gel CC eluted with petroleum ether - EtOAc (20:1 to 1:1), ODS C18 column (MeOH - H_2_O, 3:7–1:0), semi-preparative HPLC (MeOH - H_2_O, 75:25, 2 mL/min) | (Djimabi et al., 2022) |
| **55** | **isoambreinolide** | *norlabdane-type diterpenes* | *leaves* | MeOH extract  Hx fraction | silica gel column | (Tiwari et al., 2013) |
| **56** | **trisnor-*γ*-lactone** |  | *fruits* | 95% EtOH extract  EtOAc fraction | silica gel column chromatography (CC) and Sephadex LH-20, MCI  gel | (Djimabi et al., 2021) |
| **57** | **vitexifolin D** |  | *fruits* | 95% EtOH extract  EtOAc fraction | silica gel column chromatography (CC) and Sephadex LH-20, MCI  gel | (Djimabi et al., 2021) |
|  |  |  | *fruit* | MeOH extract | ultra-performance liquid chromatography coupled with Orbitrap mass spec-  trometry (UPLC-Orbitrap-MS) | (Li et al., 2020b) |
| **58** | **vitedoin B** |  | *fruits* | 95% EtOH extract | silica gel, Sephadex LH-20, and Rp-8 and Rp-18 gel column chromatography | (Gu et al., 2007) |
|  |  |  | *leaf* | Aq extract | GC and GC–MS analyses | (Elumalai et al., 2015) |
| **59** | **vitrifolin A** |  | *leaf* | Aq extract | GC and GC–MS analyses | (Elumalai et al., 2015) |
| **60** | **vitexifolin E** |  | *fruit* | MeOH extract | ultra-performance liquid chromatography coupled with Orbitrap mass spec-  trometry (UPLC-Orbitrap-MS) | (Li et al., 2020b) |
|  |  |  | *fruits* | Ace extract | silica gel column and RP-18 column chromatography | (Kiuchi et al., 2004) |
| **61** | **9,13-epoxy-16-norlabda-13*E*-en-15-al** |  | *leaves* | 95% EtOH extract  EtOAc fraction | semi-preparative HPLC, silica gel CC and RP-C18 column | (Luo et al., 2017c) |
| **62** | **negundoin C** |  | *leaves* | 95% EtOH extract  EtOAc fraction | semi-preparative HPLC, silica gel CC , MCI gel column | (Luo et al., 2017c) |
| **63** | **6*β*-acetoxy-9*α*,13-epoxy-16-norlabd-13*E*-en-15-al** |  | *fruits* | 95% EtOH Extract  EtOAc fraction | silica gel column chromatography (CC) petroleum ether - acetone (40:1 to 1:1, V/V), MCI gel CC (MeOH - H_2_O, 3:7 to 1:0, V/V), silica gel CC (petroleum ether - EtOAc, 20:1 to 1:1), Sephadex LH-20 (CHCl_3_ - MeOH, 2:3), silica gel CC (CH_2_Cl_2_ - EtOAc, 50:1 to 2:1) | (Djimabi et al., 2022) |
| **64** | **vitepyrroloid A** | *labdane diterpenoid alkaloids* | *leaves* | 95% EtOH extract  EtOAc fraction | silica gel CC, RP-C18 column, semipreparative HPLC | (Luo et al., 2017a) |
| **65** | **vitepyrroloid B** |  | *leaves* | 95% EtOH extract  EtOAc fraction | silica gel CC, RP-C18 column , semipreparative HPLC | (Luo et al., 2017a) |
| **66** | **vitepyrroloid C** |  | *leaves* | 95% EtOH extract  EtOAc fraction | silica gel CC, Sephadex LH-20 column semipreparative HPLC | (Luo et al., 2017a) |
| **67** | **vitepyrroloid D** |  | *leaves* | 95% EtOH extract  EtOAc fraction | silica gel CC, Sephadex LH-20 column, semipreparative HPLC | (Luo et al., 2017a) |
| **68** | **9*α*-hydroxy-13(14)-labden-16,15-amide** |  | *leaves* | 95% EtOH extract  EtOAc fraction | semi-preparative HPLC, silica gel CC and RP-C18 column | (Luo et al., 2017c) |
| **69** | **(3S,5S,6S,8R,9R,10S)-3,6,9-trihydroxy-13(14)-labdean-16,15-olide 3-*O*-*β*-D-glucopyranoside** | *diterpenoid glucoside* | *fruits* | 80% EtOH extract  *n*-BuOH fraction | silica gel, Sephadex LH-20 and HPLC | (Bao et al., 2018) |
| **70** | **viteagnuside A** |  | *fruits* | 80% EtOH extract  *n*-BuOH fraction | silica gel, ODS open column chromatography, and HPLC | (Bao et al., 2018) |
| **71** | **vitextrifloxide F (viterofolin F)** | *Halimane diterpenoid* | *leaves* | 95% EtOH extract  EtOAc fraction | silica gel CC and MCI gel column | (Luo et al., 2017c) |
| **72** | **vitextrifloxide G** |  | *leaves* | 95% EtOH extract  EtOAc fraction | silica gel CC and MCI gel column | (Luo et al., 2017c) |
| **73** | **vitetrifolin G** |  | *fruit* | Ace extract  EtOAc fraction | silica gel and Sephadex LH-20 column chromatography, HPLC, polymer (MCI gel CHP 20P) | (Ono et al., 2001) |
|  |  |  | *leaves* | 95% EtOH extract  EtOAc fraction | semi-preparative HPLC, silica gel CC , MCI gel column | (Luo et al., 2017c) |
| **74** | **vitextrifloxide H** |  | *leaves* | 95% EtOH extract  EtOAc fraction | semi-preparative HPLC, silica gel CC ,Sephadex LH-20 column | (Luo et al., 2017c) |
| **75** | **vitetrifolin I** |  | *fruits* | 30% Ace  CHL fraction | silica gel, MCI CHP-20P, Cosmosil 75 C18-OPN, Toyopearl HW-40F, Sephadex LH-20, Al2O3 | (Wu et al., 2009a) |
|  |  |  | *leaves* | 95% EtOH extract  EtOAc fraction | semi-preparative HPLC, silica gel CC , MCI gel column | (Luo et al., 2017c) |
| **76** | **vitetrifolin D** |  | *fruit* | Ace extract  EtOAc fraction | silica gel and Sephadex LH-20 column chromatography, HPLC, polymer (MCI gel CHP 20P) | (Ono et al., 2001) |
|  |  |  | *leaves* | 95% EtOH extract  EtOAc fraction | semi-preparative HPLC, silica gel and MCI gel CC | (Luo et al., 2017c) |
|  |  |  | *fruits* | CHL and PE extracts | vacuum liquid chromatography over silica gel H chromatography | (Li et al., 2005b) |
|  |  |  | *fruits* | 30% Ace  CHL fraction | silica gel, MCI CHP-20P, Cosmosil 75 C18-OPN, Toyopearl HW-40F, Sephadex LH-20, Al2O3 | (Wu et al., 2009a) |
|  |  |  | *fruits* | 95% EtOH extract  EtOAc fraction | silica gel column chromatography (CC) and Sephadex LH-20, MCI  gel | (Djimabi et al., 2021) |
|  |  |  | *fruits* | 95% EtOH extract | silica gel, Sephadex LH-20, and Rp-8 and Rp-18 gel column chromatography | (Gu et al., 2007) |
| **77** | **vitetrifolin E** |  | *fruit* | Ace extract  EtOAc fraction | silica gel and Sephadex LH-20 column chromatography, HPLC , polymer (MCI gel CHP 20P) | (Ono et al., 2001) |
|  |  |  | *fruits* | 30% Ace  *CHL fraction* | silica gel, MCI CHP-20P, Cosmosil 75 C18-OPN, Toyopearl HW-40F, Sephadex LH-20, Al2O3 | (Wu et al., 2009a) |
|  |  |  | *fruits* | CHL and PE extracts | vacuum liquid chromatography over silica gel H, ODS column chromatography | (Li et al., 2005b) |
|  |  |  | *leaves* | 95% EtOH extract  EtOAc fraction | semi-preparative HPLC, silica gel CC , MCI gel column | (Luo et al., 2017c) |
| **78** | **vitetrifolin F (vitexifolin F)** |  | *fruits* | 30% Ace  CHL fraction | silica gel, MCI CHP-20P, Cosmosil 75 C18-OPN, Toyopearl HW-40F, Sephadex LH-20, Al2O3 | (Wu et al., 2009a) |
|  |  |  | *leaves* | 95% EtOH extract  EtOAc fraction | semi-preparative HPLC, silica gel CC , MCI gel column | (Luo et al., 2017c) |
|  |  |  | *fruits* | Ace extract | silica gel column and RP-18 column chromatography | (Kiuchi et al., 2004) |
| **79** | **vitetrifolin H** |  | *fruits* | 80% EtOH extract  DCM fraction | silica gel and Sephadex LH20 column chromatography, semipreparative HPLC | (Zheng et al., 2013a) |
| **80** | **13-hydroxy-5(10),14-halimadien-6-one** |  | *leaves* | M eOH extract  Hx fraction | silica gel column, preparative HPLC | (Tiwari et al., 2013) |
|  |  |  | *leaves* | 95% EtOH extract  EtOAc fraction | semi-preparative HPLC, silica gel CC column | (Luo et al., 2017c) |
| **81** | **viterofolin D** |  | *fruits* | 95% EtOH Extract  EtOAc fraction | silica gel column chromatography (CC), MCI gel CC, silica gel CC, ODS C18, silica gel | (Djimabi et al., 2022) |
| **82** | **vitexfolin B** | *Clerodane diterpenoid* | *leaves* | 95% EtOH extract  EtOAc fraction | RP-C18 column and silica gel column | (Luo et al., 2017c) |
| **83** | **vitextrifloxide I** |  | *leaves* | 95% EtOH extract  EtOAc fraction | RP-C18 column and silica gel column | (Luo et al., 2017c) |
| **84** | **dysoxydensin G** |  | *leaves* | 95% EtOH extract  EtOAc fraction | semi-preparative HPLC, silica gel CC and MCI gel column | (Luo et al., 2017c) |
| **85** | **vitetrifolin A** | *abietane-type diterpene* | *fruits* | Ace extract  Hx fraction | silica gel column chromatography (CC) and HPLC | (Ono et al., 2000) |
|  |  |  |  | MeOH extract | ultra-performance liquid chromatography coupled with Orbitrap mass spectrometry (UPLC-Orbitrap-MS) | (Li et al., 2020b) |
|  |  |  |  | Ace extract  EtOAc fraction | silica gel and Sephadex LH-20 column chromatography, HPLC , polymer (MCI gel CHP 20P) | (Ono et al., 2001) |
| **86** | **abietatrien-3*β*-ol** |  | *fruit* | *Ace extract*  Hx fraction | silica gel column chromatography and ODS-HPLC | (Ono et al., 2000) |
|  |  |  |  | *95% EtOH extract*  *EtOAc fraction* | silica gel column chromatography | (Djimabi et al., 2021) |
|  |  |  |  | 95% EtOH extract | silica gel, Sephadex LH-20, and Rp-8 and Rp-18 gel column chromatography | (Gu et al., 2007) |
| **87** | **ferruginol** |  | *fruit* | MeOH extract | ultra-performance liquid chromatography coupled with Orbitrap mass spec-  trometry (UPLC-Orbitrap-MS) | (Li et al., 2020b) |
| **88** | **3*β*-acetoxyabieta-8,11,13-triene-12-ol** |  | *fruits* | 95% EtOH extract  EtOAc fraction | silica gel column chromatography (CC) and Sephadex LH-20, MCI  gel | (Djimabi et al., 2021) |
| **89** | **vitexifolin C** | *Abeo-abietane-type diterpenoid* | *fruit* | MeOH extract | ultra-performance liquid chromatography coupled with Orbitrap mass spec-  trometry (UPLC-Orbitrap-MS) | (Li et al., 2020b) |
| **90** | **helipterol** | *cyclophytane-type* | *fruits* | 95% EtOH extract  EtOAc fraction | silica gel column chromatography (CC) and Sephadex LH-20 | (Djimabi et al., 2021) |
| **Triterpenoids And Phytosterol** | | | | | | |
| **91** | **oleanolic acid** | *Oleanane* *triterpenoid* | *leaves* | EtOAc extract | silica gel column chromatography and ODS-HPLC | (Nishina et al., 2017) |
|  |  |  |  | EtOH extract  *n*-butanol fraction | silica gel 60, Sephadex LH-20 column chromatography | (Mohamed et al., 2012) |
| **92** | **hederagenin** |  |  | EtOH extract  *n*-butanol fraction | silica gel 60, Sephadex LH-20 column chromatography | (Mohamed et al., 2012) |
| **93** | **2*α*, 3*β*, 24-trihydroxyolean-12-en-28-oic acid** |  | *stems and leaves* |  |  | (Liu et al., 2014) |
| **94** | **2*α*, 3*α*, 24-trihydroxyolean-12-en-28-oic acid** |  |  |  |  | (Liu et al., 2014) |
| **95** | **maslinic acid** |  | *fruit* | MeOH extract | ultra-performance liquid chromatography coupled with Orbitrap mass spec-  trometry (UPLC-Orbitrap-MS) | (Li et al., 2020b) |
| **96** | ***β*-amyrin** |  | *leaves* | EtOH extract  *n*-butanol fraction | silica gel 60, Sephadex LH-20 column chromatography | (Mohamed et al., 2012) |
| **97** | ***β*-amyrin-3-*O*-glucopyranoside** |  | *leaves* | EtOH extract  *n*-butanol fraction | silica gel 60, Sephadex LH-20 column chromatography | (Mohamed et al., 2012) |
| **98** | **23-hydroxy-3*α*-[*O*-*α*-L-rhamnopyranosyl-(1′′′→4″)-*O*-[*β*-D-(*E*-6″-*O*-caffeoyl)-glucopyranosyl]-oxy]-olean-12-en-28-oic acid** |  | *leaves* | EtOH extract  *n*-butanol fraction | Silica gel 60, Sephadex LH-20 column chromatography | (Mohamed et al., 2012) |
| **99** | **23-hydroxy-3*α*-(*O*-sulfonyloxy)-olean-12-en-28-oic acid-28-*O*-[*α*-L-rhamnopyranosyl-(1′′′→4″)-*O*-*β*-D-glucopyranosyl-(1″→6′)-*O*-*β*-D-glucopyranosyl] ester** |  |  | EtOH extract  *n*-butanol fraction | Slica gel 60, Sephadex LH-20 column chromatography | (Mohamed et al., 2012) |
| **100** | **ursolic acid** | *Ursane* *triterpenoid* | *leaves* | MeOH extract  Hx fraction | Silica gel column | (Tiwari et al., 2013) |
| **101** | **3-epiursolic acid** |  | *stems and leaves* |  |  | (Liu et al., 2014) |
| **102** | **ursolic acid acetate** |  | *leaves* | EtOH extract | Silica gel column chromatography, RP-HPLC | (Jangwan et al., 2013) |
| **103** | **2*α*,3*β*-dihydroxyurs-12-en-28-oic acid (corosolic acid)** |  | *leaves* | MeOH extract  *n*-butanol fraction | Silica gel column, preparative HPLC | (Tiwari et al., 2013) |
| **104** | **2*α*,3*α*-dihydroxyurs-12-en-28-oic acid (3-epicorosolic acid)** |  |  |  | Chromatography and spectroscopic analysis | (Chen et al., 2010) |
| **105** | ***α*-amyrin** |  | *leaves* | MeOH extract  Hx fraction | Silica gel column | (Tiwari et al., 2013) |
| **106** | **2*α*,3*β*, 19-trihydroxyurs-12-en-28-oic acid (tormentic acid)** |  |  |  | Chromatography and spectroscopic analysis | (Chen et al., 2010) |
| **107** | **uvaol** |  | *stems and leaves* |  |  | (Liu et al., 2014) |
| **108** | **2*α*, 3*α*, 24-trihydroxyurs-12-en-28-oic acid** |  | *stems and leaves* |  |  | (Liu et al., 2014) |
| **109** | **betulinic acid**  Lupane Triterpenoid |  | *fruit* | MeOH extract | Ultra-performance liquid chromatography coupled with Orbitrap mass spec-  trometry (UPLC-Orbitrap-MS) | (Li et al., 2020b) |
| **110** | **platanic acid** |  | *leaves* | EtOH extract | Silica gel column chromatography, RP-HPLC | (Jangwan et al., 2013) |
| **111** | **taraxerol**  Taraxerane Triterpenoid |  |  |  | Chromatography and spectroscopic analysis | (Chen et al., 2010) |
| **112** | **stigmasterol** | *Phytosterol* | *leaves* | EtOH extract  EtOAc fraction | Silica gel 60 column chromatography | (Mohamed et al., 2012) |
| **113** | ***β*-sitosterol** |  | *fruits* | 95% EtOH extract  EtOAc fraction | Silica gel column chromatography | (Djimabi et al., 2021) |
|  |  |  | *stems and leaves* |  |  | (Liu et al., 2014) |
|  |  |  | *leaves* | EtOH extract | Silica gel column chromatography, RP-HPLC | (Jangwan et al., 2013) |
| **114** | ***β*-daucosterol** |  | *stems and leaves* |  |  | (Zeng et al., 1996) |
| **115** | **stigmast-4-ene-3,6-dione** |  | *fruits* | 95% EtOH extract  EtOAc fraction | Silica gel column chromatography | (Djimabi et al., 2021) |
| **116** | **6*β*-hydroxystigmast-4-en-3-one** |  | *fruits* | 95% EtOH extract  EtOAc fraction | Silica gel column chromatography (CC) and MCI  gel | (Djimabi et al., 2021) |
| **117** | **stigmast-4-en-6*β*-ol-3-one** |  | *fruits* | *n*-BuOH extract | Silica gel, RP-18 and Sephadex LH-20 column chromatography | (Gu et al., 2008) |
| **118** | **3*α*-hydroxylanosta-8,24*E*-dien-26-oic acid** |  | *leaves* | DCM, EtOAc, and Aq extracts | Chromatographic methods | (Ban et al., 2018) |
| **119** | **ergosterol peroxide** | *ergostanoid* | *fruit* | MeOH extract | Ultra-performance liquid chromatography coupled with Orbitrap mass spec-  trometry (UPLC-Orbitrap-MS) | (Li et al., 2020b) |
|  |  |  | *fruits* | 95% EtOH extract | Silica gel, Sephadex LH-20, and Rp-8 and Rp-18 gel column chromatography | (Gu et al., 2007) |
| **120** | **ecdysone** | *phytoecdysteroids* | *leaves* | DCM, EtOAc, and Aq extracts | Chromatographic methods | (Ban et al., 2018) |
|  |  |  | *leaves* | Hot MeOH extract  EtOAc fraction | Silica gel, RP-18, Sephadex LH-20 column | (Thoa et al., 2018) |
| **121** | **20-hydroxyecdysone** |  | *leaves* | DCM, EtOAc, and Aq extracts | Chromatographic methods | (Ban et al., 2018) |
|  |  |  | *leaves* | Aq extract | GC and GC–MS analyses | (Elumalai et al., 2015) |
|  |  |  | *leaves* | Hot MeOH extract  EtOAc fraction | Silica gel, RP-18, Sephadex LH-20 column | (Thoa et al., 2018) |
| **122** | **20-hydroxyecdysone 2,3-monoacetonide** |  | *leaves* | DCM, EtOAc, and Aq extracts | Chromatographic methods | (Ban et al., 2018) |
|  |  |  | *leaves* | Hot MeOH extract  EtOAc fraction | Silica gel, RP-18, Sephadex LH-20 column | (Thoa et al., 2018) |
| **123** | **turkesterone** |  | *leaves* | DCM, EtOAc, and Aq extracts | Chromatographic methods | (Ban et al., 2018) |
|  |  |  | *leaves* | Hot MeOH extract  Aq fraction | Silica gel, Diaion HP-20P, RP-18 column | (Thoa et al., 2018) |
| **124** | **polypodine B** |  | *leaves* | DCM, EtOAc, and Aq extracts | Chromatographic methods | (Ban et al., 2018) |
| **Flavonoids** | | | | | | |
| **125** | **apigenin** | *flavone* | *fruit* | MeOH extract | Ultra-performance liquid chromatography coupled with Orbitrap mass spectrometry (UPLC-Orbitrap-MS) | (Li et al., 2020b) |
| **126** | **vitexin** |  | *leaves* | MeOH extract  *n*- BuOH fraction | Silica gel column, preparative HPLC | (Tiwari et al., 2013) |
|  |  |  | *fruit* | MeOH extract | Ultra-performance liquid chromatography coupled with Orbitrap mass spectrometry (UPLC-Orbitrap-MS) | (Li et al., 2020b) |
| **127** | **luteolin** |  | *fruits* | 95% EtOH extract  CHL fraction | Vacuum liquid chromatography over silica gel H  Sephadex LH-20 column, polyamide column chromatography | (Li et al., 2005a) |
|  |  |  | *fruit* | MeOH extract | Ultra-performance liquid chromatography coupled with Orbitrap mass spectrometry (UPLC-Orbitrap-MS) | (Li et al., 2020b) |
| **128** | **luteolin-7-glucuronide** |  | *fruit* | MeOH extract | Ultra-performance liquid chromatography coupled with Orbitrap mass spectrometry (UPLC-Orbitrap-MS) | (Li et al., 2020b) |
| **129** | **orientin** |  | *fruit* | MeOH extract | Ultra-performance liquid chromatography coupled with Orbitrap mass spectrometry (UPLC-Orbitrap-MS) | (Li et al., 2020b) |
| **130** | **cynaroside** |  | *fruit* | MeOH extract | Ultra-performance liquid chromatography coupled with Orbitrap mass spectrometry (UPLC-Orbitrap-MS) | (Li et al., 2020b) |
| **131** | **isoorientin** |  | *fruit* | MeOH extract | Ultra-performance liquid chromatography coupled with Orbitrap mass spectrometry (UPLC-Orbitrap-MS) | (Li et al., 2020b) |
| **132** | **5-hydroxy-3',4',6,7-tetramethoxyflavone** |  | *fruit* | MeOH extract | Ultra-performance liquid chromatography coupled with Orbitrap mass spectrometry (UPLC-Orbitrap-MS) | (Li et al., 2020b) |
| **133** | **quercetin** | *flavonol* | *fruit* | MeOH extract | Ultra-performance liquid chromatography coupled with Orbitrap mass spectrometry (UPLC-Orbitrap-MS) | (Li et al., 2020b) |
| **134** | **quercetin 7-*O*-neohesperidoside** |  | *leaves* | EtOH extract  EtOAc fraction | Silica gel 60, Sephadex column chromatography | (Mohamed et al., 2012) |
| **135** | **vitexicarpin (casticin)** |  | *leaves* | CHL extract | Silica gel column chromatography | (NAIR et al., 1975) |
|  |  |  |  |  | High-speed counter-current chromatography | (Meng et al., 2006) |
|  |  |  | *leaves* | MeOH extract  CHL fraction | Silica gel column, preparative TLC | (Tiwari et al., 2013) |
|  |  |  | *aerial parts* | MeOH extract  CHL fraction | Silica gel column chromatography | (Tiwari et al., 2011) |
|  |  |  | *leaves* | HX extract  Ace fraction | Preparative TLC | (Alam et al., 2002) |
|  |  |  | *fruits* | 95% EtOH extract  EtOAc fraction | Silica gel column chromatography (CC) and MCI gel | (Djimabi et al., 2021) |
|  |  |  | *seeds* |  | High-speed counter-current chromatography (HSCCC) method, HPLC | (Guan et al., 2010) |
|  |  |  | *fruits* | 95% EtOH extract  CHL fraction | Vacuum liquid chromatography over silica gel H Sephadex LH-20 column | (Li et al., 2005a) |
|  |  |  | *leaves* | EtOAc extract | Silica gel column chromatography, ODS-HPLC | (Nishina et al., 2017) |
|  |  |  | *fruit* | MeOH extract | Ultra-performance liquid chromatography coupled with Orbitrap mass spectrometry (UPLC-Orbitrap-MS) | (Li et al., 2020b) |
|  |  |  | *fruits* | 95% EtOH extract | Silica gel, Sephadex LH-20, and Rp-8 and Rp-18 gel column chromatography | (Gu et al., 2007) |
| **136** | **3,4'-dimethoxy quercetin 7-*O*-glucopyranoside** |  | *leaves* | EtOH extract  EtOAc fraction | Silica gel 60, Sephadex column chromatography | (Mohamed et al., 2012) |
| **137** | **3,6,4'-trimethoxy quercetin 7-*O*-glucopyuranoside** |  | *leaves* | EtOH extract  EtOAc fraction | Silica gel 60, Sephadex column chromatography | (Mohamed et al., 2012) |
| **138** | **artemetin** |  | *leaves* | CHL extract | Silica gel column chromatography | (NAIR et al., 1975) |
|  |  |  | *seeds* |  | High-speed counter- current chromatography (HSCCC) method, HPLC | (Guan et al., 2010) |
|  |  |  | *fruits* | 95% EtOH extract  CHL fraction | Vacuum liquid chromatography over silica gel H  Sephadex LH-20 column | (Li et al., 2005a) |
|  |  |  | *fruit* | MeOH extract | Ultra-performance liquid chromatography coupled with Orbitrap mass spectrometry (UPLC-Orbitrap-MS) | (Li et al., 2020b) |
| **139** | **7-desmethyl artemetin** |  | *leaves* | CHL extract | silica gel column chromatography | (NAIR et al., 1975) |
| **140** | **chrysoplenol D** |  | *leaves* | MeOH extract  CHL fraction | Silica gel column, preparative TLC | (Tiwari et al., 2013) |
|  |  |  | *aerial parts* | MeOH extract  CHL fraction | Silica gel column chromatography | (Tiwari et al., 2011) |
|  |  |  | *seeds* |  | high-speed counter- current chromatography (HSCCC) method, HPLC | (Guan et al., 2010) |
|  |  |  | *fruits* | 95% EtOH extract  CHL fraction | Vacuum liquid chromatography over silica gel H  Sephadex LH-20 column | (Li et al., 2005a) |
|  |  |  | *fruit* | MeOH extract | Ultra-performance liquid chromatography coupled with Orbitrap mass spec-  trometry (UPLC-Orbitrap-MS) | (Li et al., 2020b) |
| **141** | **penduletin** |  | *fruits* | 95% EtOH extract  CHL fraction | Vacuum liquid chromatography over silica gel H Sephadex LH-20 column | (Li et al., 2005a) |
| **142** | **quercetin 3-*O*-*α*-L-rhamnopyranoside** |  | *aerial parts* | hot 80% MeOH  H_2_O fraction | Polyamide, cellulose column, Sephadex LH-20 column | (Salah et al., 2012) |
| **143** | **persicogenin** | *flavanone* | *fruits* | 95% EtOH extract  CHL fraction | Vacuum liquid chromatography over silica gel H | (Li et al., 2005a) |
|  |  |  | *fruit* | MeOH extract | Ultra-performance liquid chromatography coupled with Orbitrap mass spec-  trometry (UPLC-Orbitrap-MS) | (Li et al., 2020b) |
| **144** | **3',4',5-hydroxy-7-methoxy-flavanone** |  | *fruit* | MeOH extract | Ultra-performance liquid chromatography coupled with Orbitrap mass spectrometry (UPLC-Orbitrap-MS) | (Li et al., 2020b) |
| **Lignans, Phenylpropanoids And** **Xanthones** | | | | | | |
| **145** | **matairesinol 4′-*O*-*β*-D- glucopyranoside** | *lignan* | *leaves* | DCM, EtOAc, and Aq extracts | chromatographic methods | (Ban et al., 2018) |
| **146** | **2-(3,4-dihydroxyphenyl)ethyl-2-*O*-[6-deoxy-*α*-L-rhmannopyranosyl-4-(3,4-dihydroxyphenyl)- 2-**  **propenoate]-*β*-D-glucopyranoside** |  | *aerial parts* | hot 80% MeOH  H_2_O fraction | Polyamide, Sephadex LH-20 column | (Salah et al., 2012) |
| **147** | **(-)-hinokinin** |  | *leaves* | MeOH extract  EtOAc fraction | Silica gel medium pressure column chromatography (MPLC), octa decyl silyl (ODS) column MPLC, high-pressure liquid chromatography (HPLC) | (Ukiya et al., 2019b) |
| **148** | **(-)-cubebin** |  | *leaves* | MeOH extract  EtOAc fraction | Silica gel medium pressure column chromatography (MPLC), octadecyl silyl (ODS) column MPLC, high-pressure liquid chromatography (HPLC) | (Ukiya et al., 2019b) |
| **149** | **(-)-*O*-methylcubebin (Mc)** |  | *leaves* | MeOH extract  EtOAc fraction | Silica gel medium-pressure column chromatography (MPLC), octadecyl silyl (ODS) column MPLC, high-pressure liquid chromatography (HPLC) | (Ukiya et al., 2019b) |
| **150** | **detetrahydroconidendrin** |  | *fruit* | MeOH extract | ultra-performance liquid chromatography coupled with Orbitrap mass spectrometry (UPLC-Orbitrap-MS) | (Li et al., 2020b) |
| **151** | **vitedoin A** | *Phenyldihydronaphthalene lignan* | *fruit* | MeOH extract | ultra-performance liquid chromatography coupled with Orbitrap mass spectrometry (UPLC-Orbitrap-MS) | (Li et al., 2020b) |
| **152** | **vitrofolal A** | *norlignan* | *fruit* | MeOH extract | ultra-performance liquid chromatography coupled with Orbitrap mass spectrometry (UPLC-Orbitrap-MS) | (Li et al., 2020b) |
| **153** | **paulownin** | *furofuran lignan* | *fruits* | 95% EtOH extract | Silica gel, Sephadex LH-20, and Rp-8 and Rp-18 gel column chromatography | (Gu et al., 2007) |
| **154** | **vitrifol A** | *dimer of*  *dihydro benzofuran neolignan* | *fruits* | *n*-BuOH extract | Silica gel column chromatography | (Gu et al., 2008) |
| **155** | **dihydrodehydrodiconifenyl alcohol** | *Neolignan* | *fruits* | *n*-BuOH extract | Silica gel column chromatography | (Gu et al., 2008) |
| **156** | **chlorogenic acid** | *Phenylpropanoids* | *fruit* | MeOH extract | Ultra-performance liquid chromatography coupled with Orbitrap mass spec-  trometry (UPLC-Orbitrap-MS) | (Li et al., 2020b) |
| **157** | **conifer aldehyde** |  | *fruits* | 95% EtOH extract  EtOAc fraction | Silica gel column chromatography (CC) and Sephadex LH-20, MCI gel, preparative TLC | (Djimabi et al., 2021) |
| **158** | **caffeic acid** |  | *leaves* | EtOH extract  EtOAc fraction | Silica gel 60 column chromatography | (Mohamed et al., 2012) |
| **159** | ***E*-methyl caffeate** |  | *aerial parts* | hot 80% MeOH  H_2_O fraction | Polyamide, cellulose column, Sephadex LH-20 column | (Salah et al., 2012) |
| **160** | **diaporxanthone G** | *unusual xanthone monomer* | *fungi Diaporthe goulteri L17 isolated from*  *fresh fruit* | EtOAc crude  extract | column chromatography on silica gel, semi-preparative HPLC | (Peng et al., 2021) |
| **161** | **diaporxanthone A** | *xanthone dimer* | fungi *Diaporthe goulteri* L17 isolated from  *fresh fruit* | EtOAc crude  extract | column chromatography on silica gel, semi-preparative HPLC, Flash chromatography | (Peng et al., 2021) |
| **162** | **diaporxanthone B** |  | *fungi Diaporthe goulteri L17 isolated from*  *fresh fruit* | EtOAc crude  extract | column chromatography on silica gel, semi-preparative HPLC | (Peng et al., 2021) |
| **163** | **diaporxanthone C** |  | *fungi Diaporthe goulteri L17 isolated from*  *fresh fruit* | EtOAc crude  extract | column chromatography on silica gel, semi-preparative HPLC | (Peng et al., 2021) |
| **164** | **diaporxanthone D** |  | *fungi Diaporthe goulteri L17 isolated from*  *fresh fruit* | EtOAc crude  extract | column chromatography on silica gel, semi-preparative HPLC | (Peng et al., 2021) |
| **165** | **diaporxanthone E** |  | *fungi Diaporthe goulteri L17 isolated from*  *fresh fruit* | EtOAc crude  extract | column chromatography on silica gel, semi-preparative HPLC | (Peng et al., 2021) |
| **166** | **diaporxanthone F** |  | *fungi Diaporthe goulteri L17 isolated from*  *fresh fruit* | EtOAc crude  extract | column chromatography on silica gel, semi-preparative HPLC | (Peng et al., 2021) |
| **167** | **dicerandrol B** |  | *fungi Diaporthe goulteri L17 isolated from*  *fresh fruit* | EtOAc crude  extract | column chromatography on silica gel, semi-preparative HPLC | (Peng et al., 2021) |
| **168** | **phomoxanthone E** |  | *fungi Diaporthe goulteri L17 isolated from*  *fresh fruit* | EtOAc crude  extract | column chromatography on silica gel, semi-preparative HPLC | (Peng et al., 2021) |
| **169** | **deacetylphomoxanthone C** |  | *fungi Diaporthe goulteri L17 isolated from*  *fresh fruit* | EtOAc crude  extract | column chromatography on silica gel, semi-preparative HPLC | (Peng et al., 2021) |
| **170** | **12-*O*-deacetyl-phomoxanthone A** |  | *fungi Diaporthe goulteri L17 isolated from*  *fresh fruit* | EtOAc crude  extract | column chromatography on silica gel, semi-preparative HPLC | (Peng et al., 2021) |
| **171** | **phomoxanthone A** |  | *fungi Diaporthe goulteri L17 isolated from*  *fresh fruit* | EtOAc crude  extract | column chromatography on silica gel, semi-preparative HPLC | (Peng et al., 2021) |
| **172** | **penexanthone A** |  | *fungi Diaporthe goulteri L17 isolated from*  *fresh fruit* | EtOAc crude  extract | column chromatography on silica gel, semi-preparative HPLC | (Peng et al., 2021) |
| **173** | **phomolactonexanthone A** |  | *fungi Diaporthe goulteri L17 isolated from*  *fresh fruit* | EtOAc crude  extract | column chromatography on silica gel, semi-preparative HPLC | (Peng et al., 2021) |
| **Other Metabolites** | | | | | | |
| **174** | **physcion** | *anthraquinone* | *fruits* | 95% EtOH extract | Silica gel, Sephadex LH-20, and Rp-8 and Rp-18 gel column chromatography | (Gu et al., 2007) |
| **175** | **2-hydroxy, 3-methoxy benzoic acid** | *benzoic acids and their derivatives* | *leaves* | MeOH extract  *n*- BuOH fraction | Silica gel column | (Tiwari et al., 2013) |
| **176** | **2,3 dihydroxy benzoic acid** |  | *leaves* | MeOH extract  *n*- BuOH fraction | Silica gel column, preparative TLC | (Tiwari et al., 2013) |
| **177** | ***p*-methoxy benzoic acid** |  | *leaves* | MeOH extract  CHL fraction | Silica gel column | (Tiwari et al., 2013) |
|  |  |  | *aerial parts* | MeOH extract  CHL fraction | Silica gel column chromatography | (Tiwari et al., 2011) |
|  |  |  | *leaves* | EtOH extract  EtOAc fraction | Silica gel 60, Sephadex LH- 20 column chromatography | (Mohamed et al., 2012) |
| **178** | ***p*-hydroxy benzoic acid** |  | *leaves* | MeOH extract  CHL fraction | Silica gel column, preparative TLC | (Tiwari et al., 2013) |
|  |  |  | *aerial parts* | MeOH extract  CHL fraction | Silica gel column chromatography | (Tiwari et al., 2011) |
|  |  |  | *seeds* |  | High-speed counter-current chromatography (HSCCC) method, HPLC | (Guan et al., 2010) |
|  |  |  | *leaves and bark* | MeOH extract  HX, CHL, EtOAc , Aq fraction | RP-18 | (Dhanani et al., 2015) |
|  |  |  | *leaves* | MeOH extract | HPLC method | (Shah et al., 2013) |
|  |  |  | *aerial parts* | MeOH extract  *n-* BuOH fraction | Silica gel 60 F254 plates | (Tiwari et al., 2012) |
|  |  |  | *fruits* | 95% EtOH extract | Silica gel, Sephadex LH-20, and Rp-8 and Rp-18 gel column chromatography | (Gu et al., 2007) |
| **179** | ***p*-hydroxybenzoic acid ethyl ester** |  | *fruits* | 95% EtOH extract  EtOAc fraction | Silica gel column chromatography (CC) and Sephadex LH-20, MCI  gel | (Djimabi et al., 2021) |
| **180** | ***p*-hydroxyacetophenone** |  | *fruits* | 95% EtOH extract  EtOAc fraction | Silica gel column chromatography (CC) and Sephadex LH-20, MCI gel, preparative TLC | (Djimabi et al., 2021) |
| **181** | **vanillin** |  | *fruits* | 95% EtOH extract  EtOAc fraction | Silica gel column chromatography (CC) and Sephadex LH-20, MCI gel, preparative TLC | (Djimabi et al., 2021) |
| **182** | **methyl-*p*-hydroxybenzoate** |  | *leaves* | MeOH extract | Silica gel column chromatography |  |
| **183** | **palmitic acid** | *saturated long-chain fatty acid* | *leaves* | MeOH: Bz: concentrated H_2_SO_4_  extract | Gas chromatography | (Kannathasan et al., 2008) |
| **184** | **1,1-bicyclopropyl-2-octanoic acid,2’-hexyl; methyl ester** |  | *leaves* | Aq extract | GC and GC–MS analyses | (Elumalai et al., 2015) |
| **185** | **oleic acid** | *unsaturated long-chain fatty acid* | *leaves* | MeOH: Bz: concentrated H_2_SO_4_  extract | Gas chromatography | (Kannathasan et al., 2008) |
| **186** | **(*z*)-9-hexadecenoic acid** |  | *stems and leaves* |  |  | (Liu et al., 2014) |
| **187** | **linolenic acid** |  | *leaves* | MeOH: Bz: concentrated H_2_SO_4_  extract | Gas chromatography | (Kannathasan et al., 2008) |
| **188** | **octacosyl alcohol** | *alcohol* | *stems and leaves* |  |  | (Liu et al., 2014) |
| **189** | **5-thio D-glucose** | *thiosugar* | *leaves* | Aq extract | GC and GC–MS analyses | (Zheng et al., 2013a) |

DCM: dichloromethane; PE: Petroleum ether; Ethanol: ETOH; Acetone: ACe; Ethyl acetate: EtOAc; Chloroform: CHL; Methanol: MeOH; Hexane: HX; Aqueous: Aq; Benzene: Bz; nd: not determined.
