# Supplementary material for: A comprehensive review of ethnomedicinal approaches, phytochemical analysis, and pharmacological potential of Vitex trifolia L
Source: Front Pharmacol. 2024 Mar 21;15:1322083. doi: 10.3389/fphar.2024.1322083 (PMC10991721; doi:10.3389/fphar.2024.1322083)
Supplement: Supplementary file 2 [file Table2.docx]

**Table S2.** Composition of *Vitex trifolia* essential oils

| **Origin** | **Analysed plant part** | **Stage of sample harvesting** | **Extraction type** | **Yield (%)** | **Major metabolites (%)** | **References** |
| --- | --- | --- | --- | --- | --- | --- |
| China (Yuanjiang) | F | F.S | thermal desorption | - | *α*-pinene, 1,8-cineole(eucalyptol), sabinene | (Zheng et al., 2013b) |
| China | Fr | - | ultrasonic assisted extraction (UAE) | 3.5% | 6-(3-acetyl-1-cyclopropen-1-yl)-3-hydroxy-6-methyl-2-heptanone, 4-(2,2,6-trimethyl-bicyclo[4.1.0] hept-1-yl)-butan-2-one, anticopalic acid | (Li et al., 2020b) |
| India | L | - | HD | 0.6 (v/w) | *β*-caryophyllene, 1,8-cineole, terpinyl acetate | (Thomas et al., 2019) |
| India | L | - | HD | 0.21 (w/w) | (*E*)-*β*-ocimene, 1,8-cineole, *α*-pinene | (Devi and Singh, 2014) |
| India (Tamil) | L | - | SD | 5 (w/w) | 1,8-cineole, sabinene, *β*-caryophyllene | (Chandrasekaran et al., 2019) |
| India | L | - | HD | 0.6% (v/w) | 5-(1-isopropenyl-4,5-dimethylbicyclo[4.3.0]nonan-5-yl)-3-methyl-2-pentenol acetate,  *β*-caryophyllene, *β-*caryophyllene oxide*,* 13-*epi*-manoyl oxide | (Karakoti et al., 2022) |
| Thailand (Bangkok) | L | autumn | SD | 0.13 (w/w) | *β*-caryophyllene, 1,8-cineole, terpinyl acetate | (Suksamrarn et al., 1991) |
| Thailand | L | - | SD | 0.16(V/W) | 1,8-cineole, terpinyl acetate, *β*-phellandrene | (Tawatsin et al., 2006) |
| Nigeria (Adamawa state) | L | summer | SD | 0.23 (v/w) | bicyclo[3.1.1]3,6,6-trimethyl-hept-2-ene (3,6,6-trimethyl-2-norpinene), *β*-caryophyllene, sabinene | (Musa et al., 2022) |
| Nigeria | S | autumn | SD | 0.088 (V/W) | sabinene, *β*-caryophyllene and *α*-pinene | (Yahaya et al., 2019) |
| Indonesia | L | - | SD | 0.0627- 0.0147 (w/w) | *cis*-ocimene, *α*- thujene, 5-isopropenyl-3,3-dimethyl-1-cyclopentene | (Arpiwi et al., 2020) |
| Indonesia | F | spring | HD | 0.041(V/W) | *β*-caryophyllene, 1,8-cineole, sabinene | (Adiyasa et al., 2014) |
| South Korea | L | summer | SD | 0.81 (w/w) | manoyl oxide, camphene, *α*-pinene | (Lee et al., 2016) |
| Germany | L & T | - | - | 0.11-0.28 (V/W) | *α*-pinene, camphene, terpiny lacetate | (Hansel et al., 1965) |
| - | L | - | - | - | *α*-pinene, linalool, terpinyl acetate, *β*-caryophyllene and caryophyllene oxide | (Pan et al., 1989) |
| Malaysia | L | - | HD | 0.025% (w/w) | viridiflorol, *β*-caryophyllene, *β*-elemene | (Zaki et al., 2022) |

AP: aerial part; C.Fr: crushed fruit; F: flower; Fr: fruit; T: twig; Fr.S: fruiting stage; F.S: flowering stage; HD: hydro-distillation; HS-SPME: headspace-solid phase micro-extraction; I.Fr: immature fruit; In: inflorescence; I.S: immature seed; L: leaf; M: microdistillation; M.S: mature seed; N.S: not specified; R: root; S: stem; SD: steam distillation; Se: seed; SPME: solid phase micro-extraction; S.S: seeding stage; U: umbel; V.S: vegetative; W.Fr: whole fruit
